# Protective antigenic sites identified in respiratory syncytial virus fusion protein reveals importance of p27 domain

**DOI:** 10.15252/emmm.202013847

**Published:** 2021-11-08

**Authors:** Jeehyun Lee, Youri Lee, Laura Klenow, Elizabeth M Coyle, Juanjie Tang, Supriya Ravichandran, Hana Golding, Surender Khurana

**Affiliations:** ^1^ Division of Viral Products Center for Biologics Evaluation and Research (CBER) FDA, Silver Spring MD USA

**Keywords:** epitope, F protein, neutralization, RSV, vaccine, Microbiology, Virology & Host Pathogen Interaction

## Abstract

Respiratory syncytial virus (RSV) vaccines primarily focused on surface fusion (F) protein are under development. Therefore, to identify RSV‐F protective epitopes, we evaluated 14 antigenic sites recognized following primary human RSV infection. BALB/c mice were vaccinated with F peptides, F proteins, or RSV‐A2, followed by rA2‐Line19F challenge. F peptides generated binding antibodies with minimal in vitro neutralization titers. However, several F peptides (including Site II) reduced lung viral loads and lung pathology scores in animals, suggesting partial protection from RSV disease. Interestingly, animals vaccinated with peptides (aa 101–121 and 110–136) spanning the F‐p27 sequence, which is only present in unprocessed F0 protein, showed control of viral loads with significantly reduced pathology compared with mock‐vaccinated controls. Furthermore, we observed F‐p27 expression on the surface of RSV‐infected cells as well as lungs from RSV‐infected mice. The anti‐p27 antibodies demonstrated antibody‐dependent cellular cytotoxicity (ADCC) of RSV‐infected A549 cells. These findings suggest that p27‐mediated immune response may play a role in control of RSV disease in vivo, and F‐p27 should be considered for inclusion in an effective RSV vaccine.

## Graphical abstract

This study identifies possible protective linear antigenic sites on the RSV F protein in a mouse RSV challenge model for development of RSV vaccine. We show that F‐p27 peptide control viral loads and reduced RSV disease *in vivo*. Therefore, F‐p27 should be included in an effective RSV vaccine.

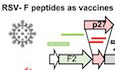

**The paper explained**

**Problem**

Respiratory syncytial virus (RSV) is the major cause of lower respiratory tract disease in infants and young children; however, there is no vaccine against RSV. RSV vaccines currently under clinical development are primarily focused on surface fusion (F) membrane protein for different target populations. However, there is limited information on antigenic sites within F that generate protection following vaccination. Therefore, it is important to identify all possible protective epitopes on the F protein for development of an effective RSV vaccine.

**Results**

In the current study, we evaluated the immunogenicity, neutralization potential, safety, and protective efficacy of all antigenic sites in F2 and F1 domains identified in the post‐primary infection infant sera using F‐GFPDL including the F‐p27 antigenic site in mouse RSV challenge model. Several F peptides provided partial protection by viral loads in lung tissues with minimal lung pathology from RSV disease. Interestingly, animals vaccinated with a peptide containing F‐p27 (aa 101–121 or 110–136), which is only present in unprocessed F0 protein, and not present on the mature RSV virion particles, showed significant control of viral loads with no apparent pathology in the lungs following RSV challenge. While p27 is not part of the mature F protein on virions, it is part of newly translated F0 in infected cells. We show for the first time that F‐p27 is expressed on the surface of RSV‐infected cells *in vitro* as well as lungs of RSV‐infected mice *in vivo*, and these cells can be killed by antibody‐dependent cell cytotoxicity, suggesting a possible role of p27‐mediated immune response in control of RSV disease.

**Impact**

The current study evaluated the immunogenicity of multiple antigenic sites within the RSV‐F protein and shows for the first time the presence of *in vivo* protective epitopes in the RSV F‐p27 motif that did not correlate with antibody binding to mature virions or neutralization *in vitro*. These findings identified p27 as a potential target of protective immunity *in vivo* and suggest inclusion of p27 in an effective vaccine against RSV.

## Introduction

Significant efforts are underway to develop and evaluate RSV vaccines targeted to pregnant women with hope of protecting neonates from RSV [renamed to human Orthopneumovirus (hOPV)]‐induced lung disease early in life, as well as to elderly populations, who are susceptible to recurrent RSV infections (Drysdale *et al*, 2020). Identification of protective epitopes in RSV proteins is critical for the development of effective vaccine against RSV disease (Johnson *et al*, 1997; Zhu *et al*, 2017; Drysdale *et al*, 2020).

In our previous study, Genome‐fragment Phage Display Libraries (GFPDL) covering the RSV‐A2 F and G genes were used to elucidate the epitope repertoires of serum antibodies from children at 9 and 18 months following primary RSV infection. Multiple sites were identified in the F protein, many of which are present in both the pre‐fusion and post‐fusion F conformations, that were confirmed by antibody binding to sera from young children (< 2 years), adolescents (14–18 years), and adults (30–45 years) (Fuentes *et al*, 2016). Surprisingly, the strongest binding in the youngest group was to peptide (aa 101–121), which contains part of p27. Expanding F‐specific epitope diversity was observed in the older groups in addition to retaining strong binding to F‐p27 (Fuentes *et al*, 2016). These findings suggested that during RSV infection, the immune system is exposed to unprocessed F0 on infected cells or to immature virions, and humans generate strong anti‐p27 response. However, the relevance of such anti‐p27 immune response in RSV disease is unknown.

One RSV vaccine candidate based on a pre‐fusogenic form of F containing p27 generated higher neutralizing antibodies compared with pre‐fusion and post‐fusion F proteins (lacking p27), and protected animals from RSV challenge in mice (Patel *et al*, 2019). In addition, this study demonstrated the generation of antibodies that competed with monoclonal antibodies (MAbs) to site Ø, II, IV, and VIII, but no evidence was provided to demonstrate the contribution of anti‐p27 antibodies to *in vitro* virus neutralization or *in vivo* protection against RSV‐A2 virus challenge (Patel *et al*, 2019). Moreover, there is limited information on antigenic sites within F that provide protection that are key for the development of an effective RSV vaccine.

In the current study, we followed up these findings through vaccination of mice with individual F‐derived peptides (representing antigenic sites identified in post‐RSV infection in humans) followed by a challenge with RSV rRSV‐A2‐L19 strain. Serum samples were tested for virion binding, RSV neutralization titers, and ADCC activity, prior to viral challenge. Lung tissues were excised on day 5 post‐challenge and evaluated for RSV viral loads, lung histopathology, and presence of CD4 and CD8 T cells. Live RSV‐A2 infection (low dose) and recombinant F proteins (pre‐fusion and post‐fusion) were used as positive controls.

## Results

### RSV‐A2 virion‐binding and neutralization antibodies following vaccination of mice with F peptides

Previously, an unbiased GFPDL analysis identified multiple antigenic sites recognized by sera from primary RSV‐infected infants that were mapped to the exposed surface of the F protein in either the pre‐fusion or post‐fusion conformation (Fuentes *et al*, 2016). In the current study, we determined their ability as an immunogen to elicit functional, virion‐binding antibodies, and the importance of these antibodies for *in vivo* protection against RSV challenge.

**Figure 1:**
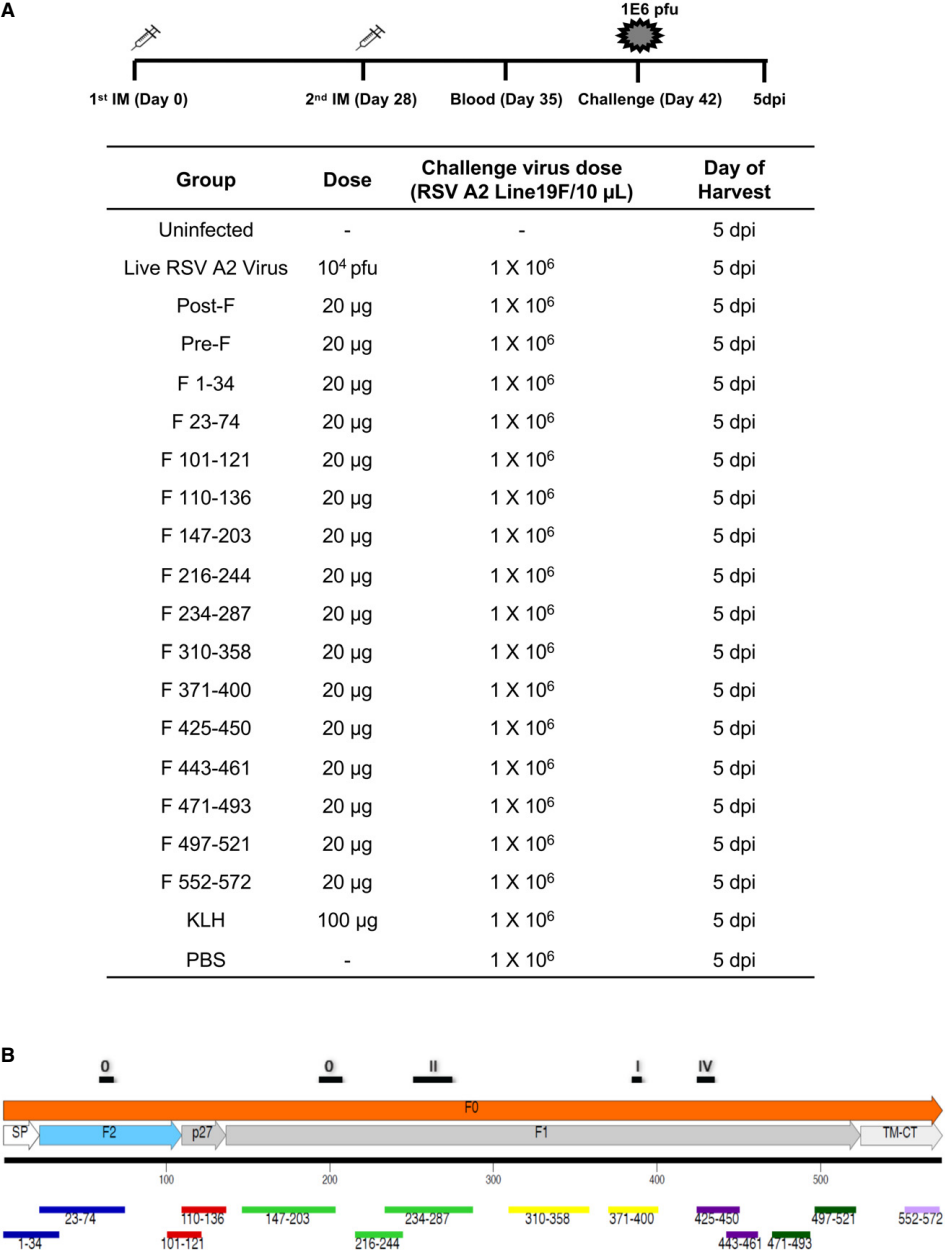
F protein immunization and challenge study in mice Schematic representation of mouse immunization and challenge schedule. Female BALB/c mice (*n* = 5 per group; 4–6 weeks old) were immunized with single dose of live RSV‐A2 virus (10^4^ pfu dose) intranasally, or two doses given i.m. with 20 μg of RSV pre‐fusion or post‐fusion form of F protein or F peptides from RSV strain A2 with Emulsigen adjuvant, or with KLH or with PBS as a control. After the second immunization, blood was collected from the tail veins on day 35. On day 42, mice were challenged intranasally with 10^6^ PFU/10μl of RSV rA2‐Line‐19F‐FFL. Mice were sacrificed on day 5 post‐challenge, when lungs and blood were collected.Alignment of antigenic site peptides within the RSV‐F protein used for mice vaccination in the study. Previously described antigenic sites (sites ϕ, I, II, and IV) are shown above the F protein schematic, and the antigenic site peptides used in this study are depicted below.

To that end, RSV‐F peptides were chemically synthesized, purified by HPLC, conjugated to KLH, and used for animal vaccination. BALB/c mice (*n* = 5 per group) were immunized intramuscularly twice with 20 μg of RSV pre‐fusion or post‐fusion forms of F protein (positive controls), or with the F peptides‐KLH conjugates mixed with Emulsigen adjuvant, or with PBS (no vaccination control), or with 100 μg of unconjugated KLH alone (carrier control), or with a single intranasal dose of 10^4^ pfu live RSV‐A2 virus (Fig 1A). The location of the different peptides in the F protein is shown in Fig 1B. The p27 is encompassed in F (aa 110–136). After the second immunization, blood was collected from the tail veins. These mice were then challenged intranasally with 1 × 10^6^ PFU/10 μl of RSV rA2‐Line‐19F‐FFL.

**Figure 2:**
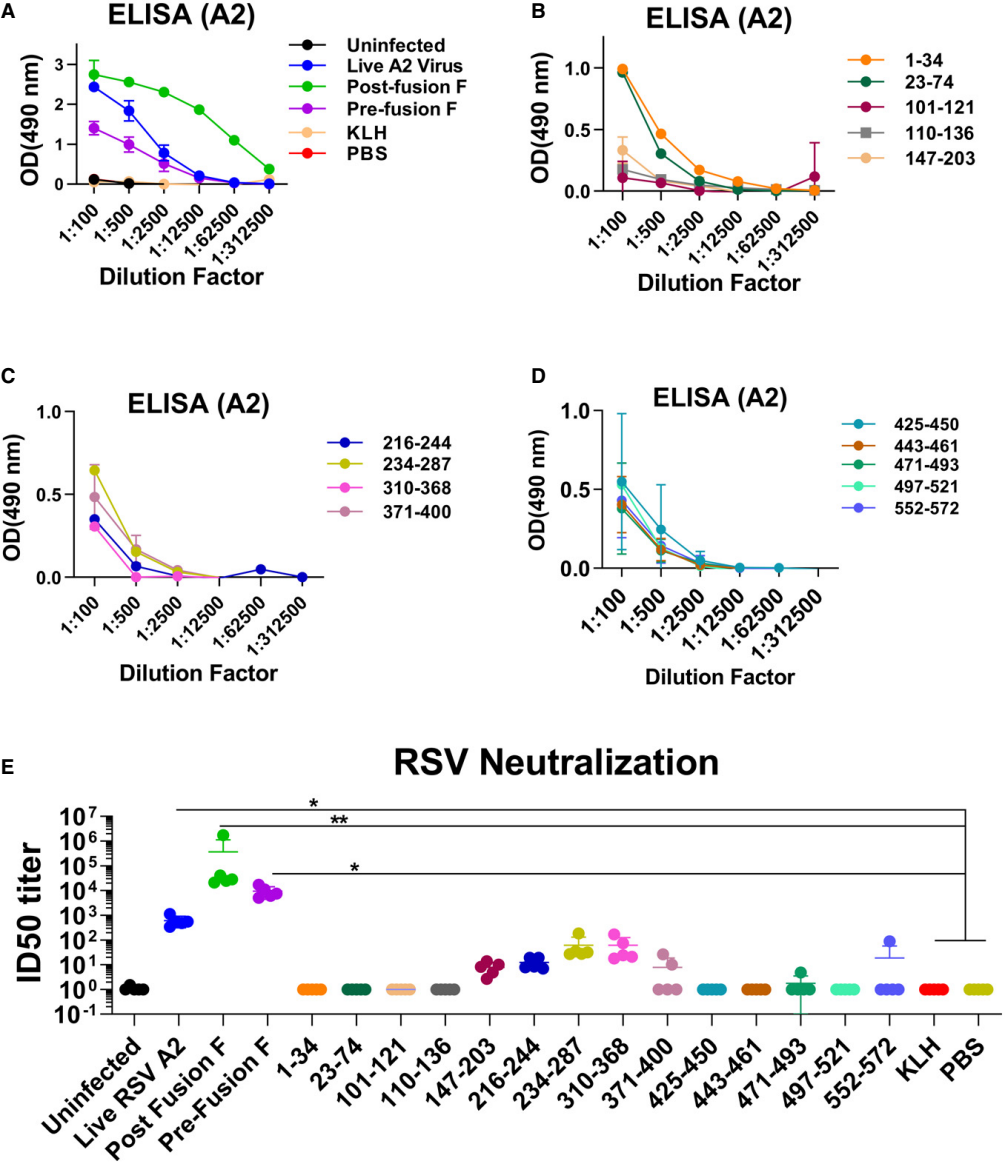
RSV binding and neutralizing antibody response following mice immunization Serum samples were collected at seven days after the second immunization from individual mice (day 35 from the start of the study; *n* = 5 mice per group) and were tested for RSV‐A2 virion binding in ELISA and neutralization by an RSV‐LINT assay against RSV‐A2 strain in A549 cells.
A–DThe serial dilutions of post‐second immunization serum samples were analyzed by ELISA using plates coated with purified RSV rA2‐Line19F‐FFL virions. The serum samples were serially diluted, and the detection of antibodies was measured by optical density (OD) at 490 nm. End‐point titers of the serum samples were determined as the reciprocal of the highest dilution providing an absorbance twice that of the negative control (PBS immunized animal).EVirus neutralization titers following second vaccination. End‐point serum dilution titer that resulted in 50% inhibition of RSV infection (ID50) in RSV‐LINT assay is shown. The mean value for each group is indicated. Data information: Two independent experiments were performed for data analysis. Results were determined and presented as mean ± SEM. Statistical significances were performed by one‐way ANOVA in GraphPad Prism; *P ≤ 0.05, **P ≤ 0.01. Source data are available online for this figure.

Post‐vaccination sera were tested for binding to RSV‐A2 virions (Fig 2A–D). The highest virus‐binding titers were observed in animals vaccinated with post‐fusion F (green curve), followed by animals exposed to live RSV‐A2 virus (blue curve), and animals vaccinated with the pre‐fusion F proteins (purple curve) (Fig 2A). No virion binding was observed with sera from either KLH‐ or PBS‐vaccinated animals Fig 2A). The individual peptides elicited antibodies with low to moderate virion binding at 100‐fold dilution with minimal to no binding observed at 1:500 following 2^nd^ vaccination (Fig 2B–D). The p27 sequence (aa 110–136) is uniquely found in uncleaved F0 and is excised during F protein maturation into F1/F2 complex and is expected to be absent on mature RSV‐A2 virion particles. Weak antibody binding to virus particle (OD of 0.15 at 100‐fold dilution) was observed for serum from mice immunized with the p27 spanning peptides 101–121 and 110–136, suggesting very minimal presence of immature or partially matured F0 proteins on virions (Fig 2B). This finding is in agreement with previous study, demonstrating that the presence of p27 peptide has a destabilizing effect on trimer formation and incorporation into virions (Krarup *et al*, 2015).

In the RSV‐LINT neutralization assay (Fuentes *et al*, 2013), high titers were measured for the three positive control groups (ID50:10^3^–10^6^). Surprisingly, several F peptides elicited detectable neutralizing activity (aa 147–203, 216–244, 234–287, 310–368) encompassing sites II (234–287) and critical contact residues for the site Ø (147–203) (Fig 2E).

### Viral loads following RSV challenge of F‐peptide vaccinated animals

**Figure 3:**
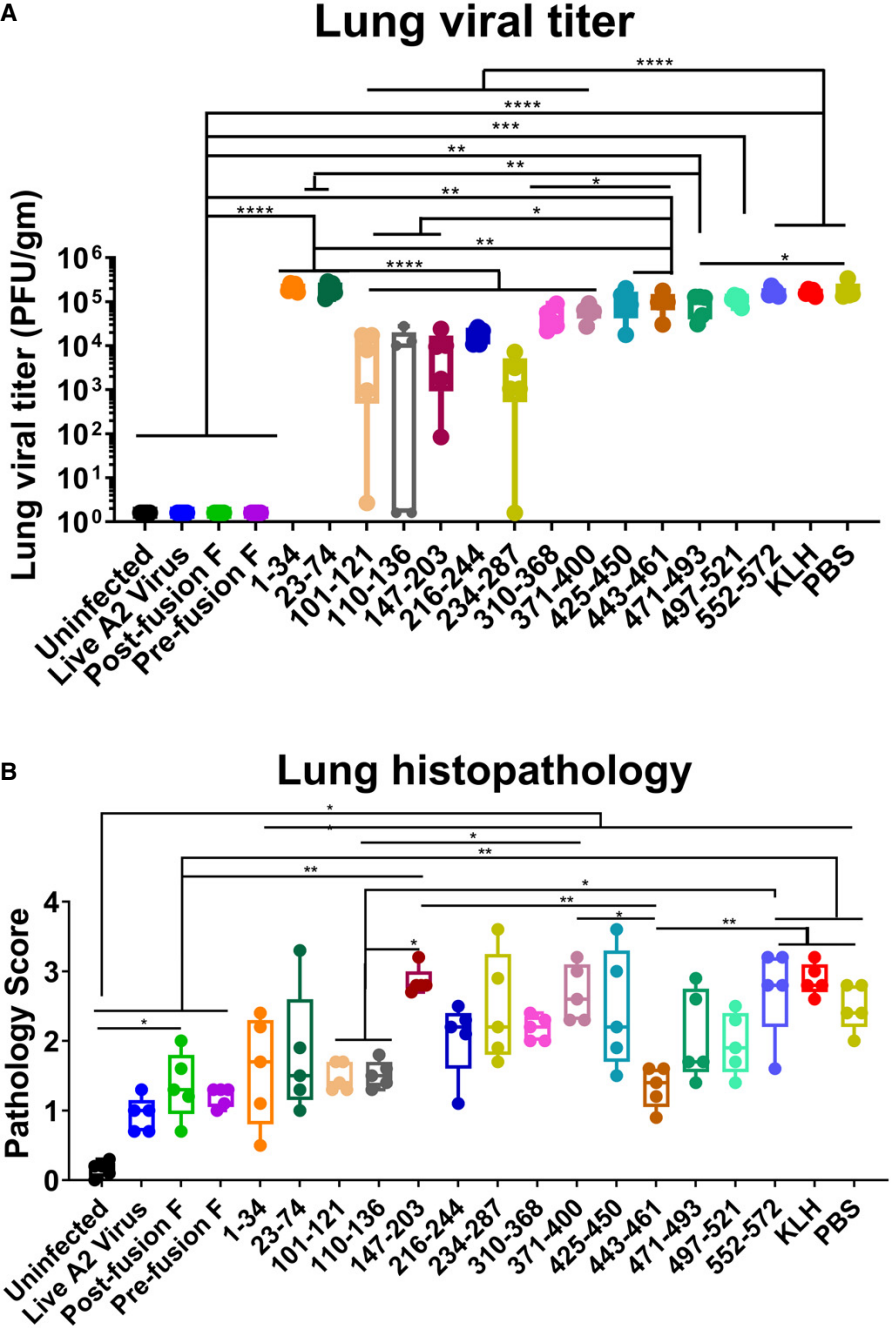
Lung viral load and histopathology of the lungs of animals vaccinated with the RSV‐F proteins and F peptides at day 5 following RSV challenge Lung RSV titers (PFU/gram of lung tissue) were determined in individual lungs (*n* = 5 mice per group) collected at 5 days post‐RSV infection. Results were determined and presented as box and whisker plots, where boxes extends from 25^th^ to 75^th^ percentile, whiskers show minimum to maximum value, and central band represents the median value for the group.Lung tissues were collected from 5 mice per group at 5 days post‐challenge and stained with hematoxylin and eosin to assess histopathology for peribronchiolar and alveolar pneumonia. Individual lungs were scored for pulmonary inflammation: bronchiolitis (mucous metaplasia of bronchioles), perivasculitis (inflammatory cell infiltration around the small blood vessels), interstitial pneumonia (inflammatory cell infiltration and thickening of alveolar walls), and alveolitis (cells within the alveolar spaces). Slides were scored blindly using a 0–4 severity scale for the pathology. The scores were subsequently converted to a combined 0–4 histopathology scales. Results are presented as box and whisker plots, where boxes extends from 25^th^ to 75^th^ percentile, whiskers show minimum to maximum value, and central band represents the median value for the group. Data information: Statistical significances were performed by one‐way ANOVA test in GraphPad Prism. The blue asterisks show significance compared to naïve infection (PBS) (*P ≤ 0.05, **P ≤ 0.01, ***P ≤ 0.001, ****P ≤ 0.0001). Source data are available online for this figure.

On day 42, mice were challenged intranasally (i.n.) with 1 × 10^6^ PFU of rRSV‐A2‐L19‐FFL as previously described (Fuentes *et al*, 2017). All animals (and KLH or PBS controls) were sacrificed on day 5 post‐infection, and the lungs were used for the measurement of viral loads (in HEp‐2 cells) and pathology scoring as outlined in Methods. Animals vaccinated with the live RSV‐A2 or the two forms of the F protein did not show measurable lung viral loads (< 5 pfu/gm lung tissue). In comparison with the PBS (control) and KLH (control), mice vaccinated with the F peptide‐KLH conjugates showed either no reduction in viral loads (aa 1–34, 23–74, and 552–572), a partial reduction in viral loads (aa 310–368, 371–400, 425–450, 443–461, 471–493, and 497–521), or significant reduction (> 90% reduction; 2 × 10^5^ pfu/gm lung in PBS controls vs. 9 × 10^3^–2 × 10^4^ pfu/gm lung in mice given F peptides) (aa 101–121, 110–136, 147–203, 216–244, and 234–287) (Fig 3A). Surprisingly, animals vaccinated with peptide 101–121, which contains a portion of the p27 peptide, and the p27 110–136 peptide, also had very low viral loads, in the absence of significant virion‐binding or *in vitro* RSV‐neutralizing antibodies (Fig 2B,E).

### Lung histopathology following RSV challenge

Several studies demonstrated that vaccine candidates based on recombinant F proteins and peptides may lead to enhanced lung pathology following vaccination of mice and cotton rats (Murphy *et al*, 1990; Connors *et al*, 1992; Lee *et al*, 2017). Therefore, at day 5 post‐RSV infection, lung tissues were histostained and subjected to blinded pathology scoring (8 fields examined from both left and right lobe per animal) as described in Methods. Lungs from animals vaccinated with live RSV‐A2 virus, or purified pre‐ and post‐fusion RSV‐F proteins, that did not have any virus replication in the lungs also displayed significantly lower histopathology, including alveolitis, bronchiolitis, perivascular, and interstitial pneumonia, compared with mock‐vaccinated (PBS) RSV‐infected mice (Fig 3B). In addition, among the peptide‐immunized animals, mice vaccinated with F peptides 101–121, 110–136 (p27), and 443–461 demonstrated significantly reduced lung pathology compared with either PBS‐ or KLH‐vaccinated animals. The differences in pathology score between RSV‐A2 intranasally immunized group vs F‐p27 vaccinated group were not significant. Mean pathology scores observed for groups given other KLH‐conjugated peptides were similar to or higher (aa147–203) than the mean pathology scores observed for the KLH or PBS mock‐immunized group, but the differences did not reach statistical significance. Interestingly, peptides 1–34 and 23–74 elicited binding antibodies that did not neutralize the virus *in vitro* (Fig 1B). Yet, the lung pathology scores for these groups were highly variable and did not reach statistical significance compared with other groups (Fig 3B). Altogether, we did not find evidence for enhanced lung pathology following challenge in any of the vaccinated groups at this antigen dose.

### F‐p27 is expressed on the surface of RSV‐infected cells and in the lungs of RSV‐infected mice

**Figure 4:**
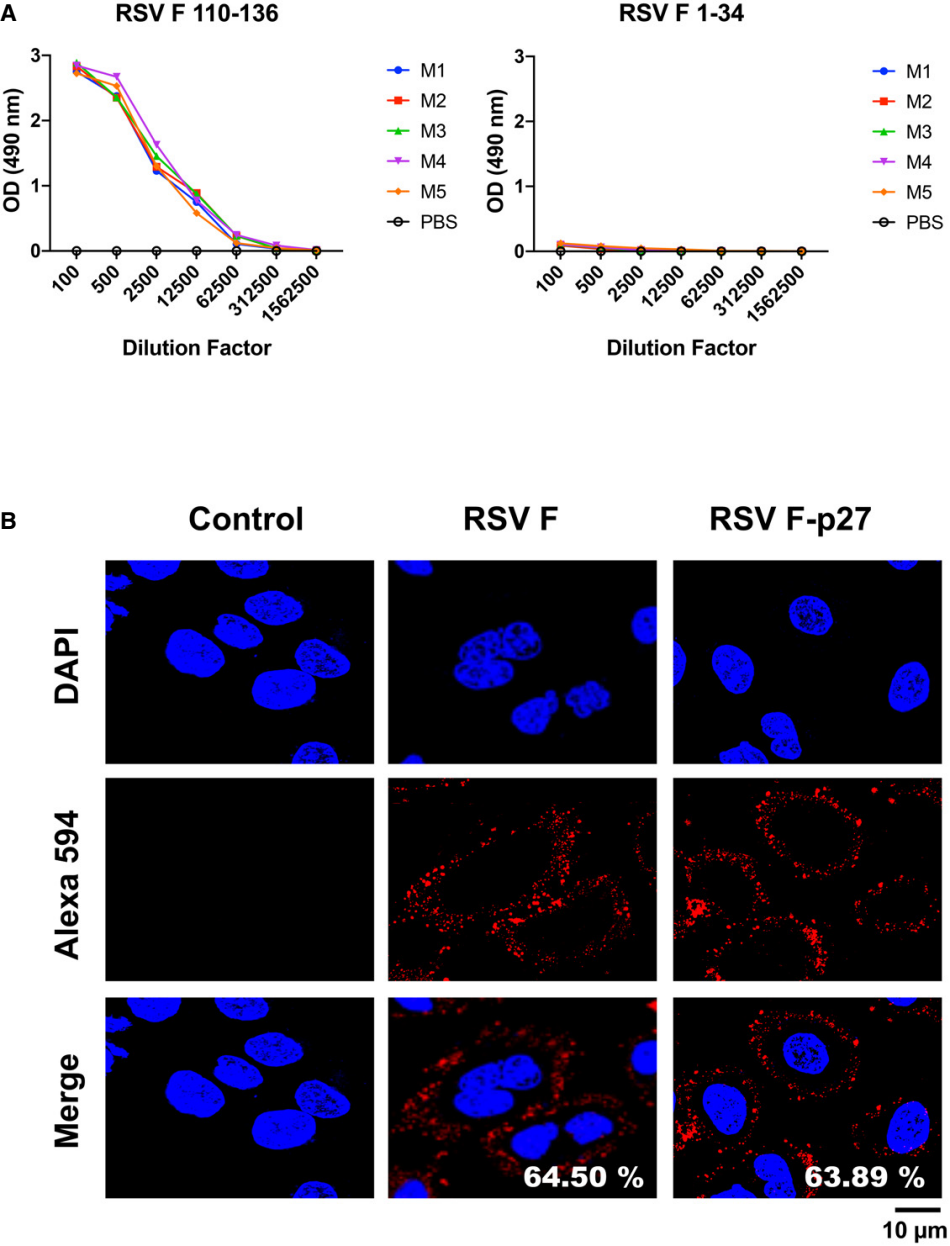
Expression of p27 on the surface of RSV‐infected A549 cells Serum samples collected from individual mouse (M 1‐5) immunized with F 110‐136 peptide were tested for antibody binding against F‐p27 (110–136) peptide or F 1‐34 peptide (control) in ELISA.A549 cells were infected with RSV (MOI = 0.1) for 16 h, and fixed. Cells were treated with mock control rabbit sera (left panels), or rabbit antisera against F (center panels), or against F‐p27 (110–136) peptide (right panels), followed by Alexa 594 conjugated anti‐Rabbit IgG (red). Nuclei are stained with DAPI (blue). Scale bar = 10 µm. The number of cells positive for RSV‐F (middle panel) and RSV F‐p27 (right panel) upon counting of 200 cells were used to calculate percentage of cells stained for each antibody staining are shown in the ‘merge’ panel. The experiments were performed twice, and variation between the two independent experiments was < 6%. Source data are available online for this figure.

While p27 (residues 110–136) is not part of the mature F protein on virions, some immature or unprocessed F0 may be present on virions (Krzyzaniak *et al*, 2013) and might not be proteolytically activated until it reaches the target cells. On the other hand, p27 is part of newly translated F0 in RSV‐infected cells. As can be seen in Fig 4A, all mice vaccinated with the p27‐containing peptide (110–136 residues) generated p27 peptide (110–136) antibody binding titers in ELISA but did not bind to F (1–34) peptide (negative control). Antisera from animals given “pre‐F” or “post‐F” proteins lacking the p27 sequence did not react to the p27 (110–136) peptide in ELISA.

To determine whether the p27 peptide is expressed on the surface of RSV‐infected cells, we generated rabbit antiserum against F protein and against F peptide containing residues 110–136 of p27 sequence. Immunostaining performed on non‐permeabilized RSV‐A2‐infected A549 cells demonstrated a comparable strong cell surface staining patterns using either anti‐RSV‐F protein or anti‐F‐p27 peptide antisera. Both antisera stained similar percentages of RSV‐A2‐infected A549 cells (64.5% and 63.89%, respectively) (Fig 4B).

**Figure 5:**
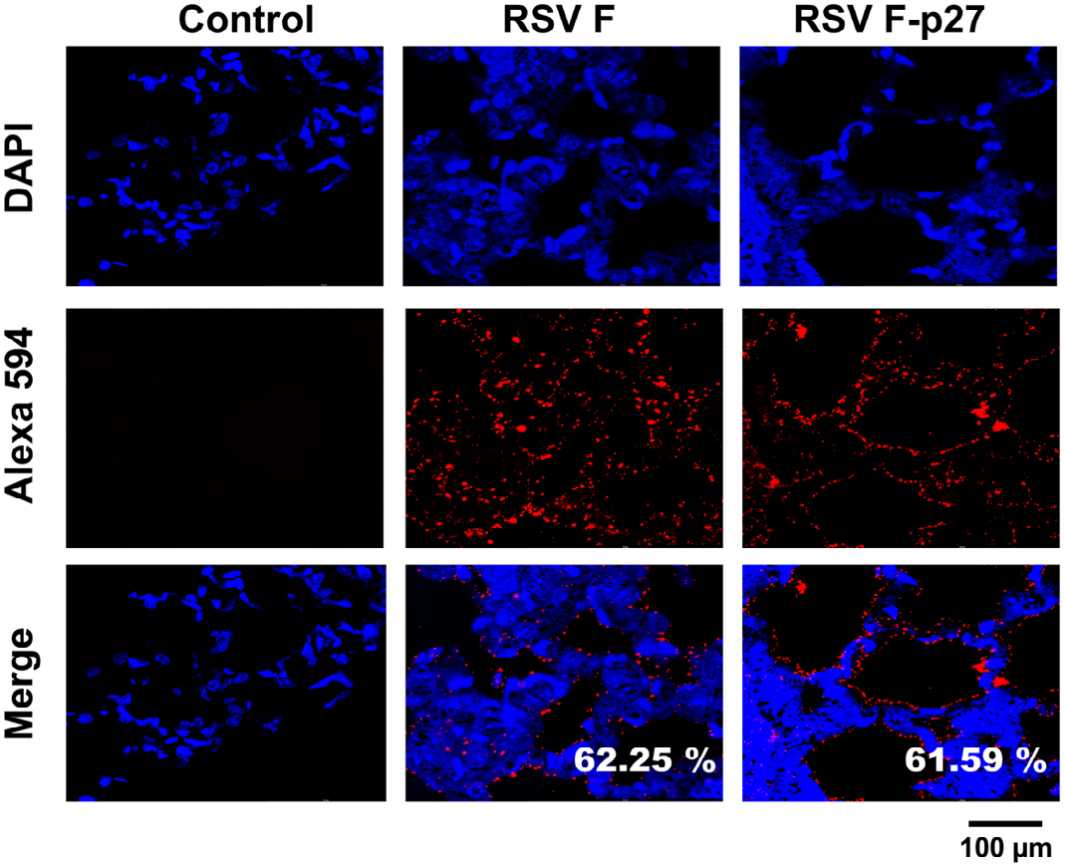
Localization of RSV antigen expression in distal alveoli in mouse lungs following viral challenge Mice lungs were collected from PBS‐(mock) vaccinated BALB/c mice at day 5 post‐RSV infection, fixed, and paraffin‐embedded, and slides were processed for imaging. Immunohistochemistry staining of lung sections shows RSV protein localized to distal alveoli by Control rabbit sera (left panels) or rabbit antisera against RSV‐F protein (center panels), or against RSV F‐p27 (110–136) peptide (right panels). Scale bar = 100 µm. The percentage of cells positive for RSV‐F (middle panel) and RSV F‐p27 (right panel) covering 500 × 500 µm of lung tissue for each mice for each antibody staining are shown. The experiments were performed twice, and variation between the two independent experiments was < 9%.

Furthermore, we also observed strong F‐p27 expression in sections of lung tissues from RSV‐A2‐infected animals using rabbit serum generated against either RSV‐F protein (62.25%) or F‐p27 (110–136) peptide (61.59%) (Fig 5). These observations suggest that F‐p27‐containing immature F0 is widely expressed on surface of RSV‐infected cells *in vitro* and in RSV‐infected lungs *in vivo*.

### Role of cell‐mediated immunity and antibody Fc effector function in anti‐p27‐mediated protection from RSV disease

**Figure 6:**
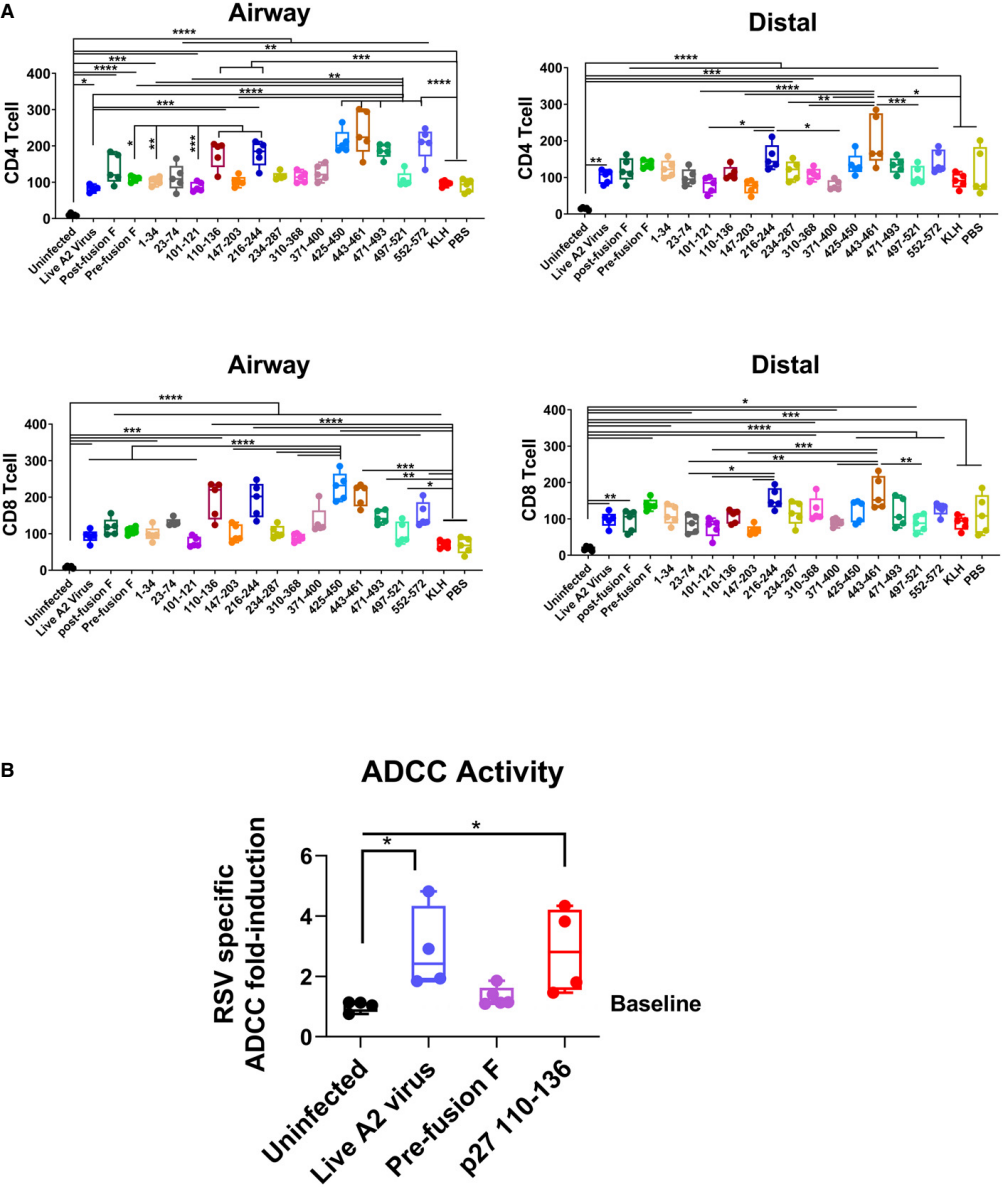
CD4/CD8 T cells in the airways and distal alveoli of mouse lungs following viral challenge and ADCC activity of post‐immunization serum Lung tissues from individual mice (*n* = 5) at day 5 post‐RSV challenge (or uninfected control) were used to evaluate CD4/CD8 T cells separately for airway and distal lungs. Lung slides were fixed and stained with DAPI to visualize nuclei (blue) and either anti‐CD4 (green) or anti‐CD8 (red) T cells. The number of CD4/CD8 T cells were determined in each lung airway and distal region. Individual CD4^+^/CD8^+^ T cells’ data are presented as number of positive cells per 174 × 174 µm of lung tissue for each mice. Results are presented as box and whisker plots, where boxes extends from 25^th^ to 75^th^ percentile, whiskers show minimum to maximum value, and central band represents the median value for the group.RSV‐specific antibody‐dependent cellular cytotoxicity (ADCC) activity of post‐second immunization serum was evaluated using RSV‐infected A549 cells or mock A549 cells that served as target cells. After incubation with serum, Jurkat/NFAT‐luc^+^ FcγRIV that expresses a luciferase reporter were added as the effector cells. The amount of luciferase was then measured as relative luciferase units (RLU). The RLU readings of ADCC induction from serum of each group were normalized to assay control (no serum) background. The RSV‐specific ADCC activity (RLU measured) fold‐change induced is shown as the ratio of normalized RLU from RSV‐infected target cells to normalized RLU from uninfected target cells. The experiments were performed twice and variation between the two independent experiments (two biological replicates) was < 12%. Results are presented as box and whisker plots, where boxes extends from 25^th^ to 75*^th^* percentile, whiskers show minimum to maximum value, and central band represents the median value for the group. Data information: Statistical significances were performed by one‐way ANOVA in GraphPad Prism; ****P < 0.0001, ***P < 0.001, **P < 0.01, *P < 0.05. Source data are available online for this figure.

The observed reduction in lung viral titers and lung pathology after challenge of mice vaccinated with F‐p27 suggested that mechanisms other than neutralizing antibodies may play a role in protection. To evaluate number of CD4 or CD8 T cells in lungs following RSV infection, we stained sections of lungs collected from mice at day 5 post‐RSV challenge and determined CD4 and CD8 T cells in both airway and distal lung tissues. As shown in Fig 6A, in all RSV challenged mice there was influx of both CD4 and CD8 T cells compared with uninfected control animals. When compared with the PBS (unvaccinated) or KLH controls, some vaccinated animals showed higher number of either CD4 or CD8 cells, but these differences did not reach statistical significance for the p27 vaccinated group.

Antibody‐dependent cellular cytotoxicity (ADCC) may play a role in control of RSV‐mediated pathology. Therefore, to determine whether anti‐p27 antibodies mediate ADCC, post‐second immunization serum was assessed with a Promega ADCC Reporter Bioassay. A549 target cells were either mock treated or infected with RSV‐A2 virus, and incubated with post‐second immunization sera, followed by the addition of genetically engineered Jurkat T effector cells that expresses mouse FcγRIV along with a luciferase reporter driven by an NFAT‐response element (NFAT‐RE). The RSV‐specific ADCC activity induced by the serum antibodies was calculated against RSV‐infected target cells compared with mock A549 cells as baseline. Sera from uninfected mice did not induce effector cell activation above the baseline (Fig 6B). The highest level of ADCC activity was observed with sera from mice immunized with live A2 virus prior to viral challenge. Importantly, sera from two out of four mice vaccinated with p27 peptide showed ADCC activity that was higher compared with sera from mice vaccinated with pre‐fusion F, which does not contain p27 sequence (Fig 6B). Together, these studies suggest that p27 immunization induces non‐neutralizing protection mechanisms against RSV that includes Fc‐mediated killing of infected cells via ADCC and possibly T cell‐mediated effector functions.

## Discussion

Primary RSV infection in children identified antigenic sites in the pre‐fusion and post‐fusion conformation of RSV‐F protein (Fuentes *et al*, 2016). Earlier studies using F peptide‐based vaccine generated strong antibody‐binding response, but those antibodies failed to bind native F proteins or offer any protection against RSV challenge (Jaberolansar *et al*, 2017). However, the importance of the human antigenic sites identified in the F protein was not completely understood. To determine the functional relevance and contribution of these F antigenic sites in virus neutralization and *in vivo* protection from RSV disease, we followed up these findings through vaccination of mice with individual F‐derived antigenic site peptides followed by a challenge with RSV. Live RSV‐A2 infection and recombinant F proteins (pre‐fusion and post‐fusion) were used as positive controls. Virion‐binding titers following peptide vaccination were relatively modest (> 150‐fold lower than the positive controls) (Fig 2A–D). The low binding of anti‐p27 peptides to virions is explained by the fact that p27 is uniquely found in uncleaved F0, which is normally excised during F protein maturation into F1/F2 complex and is expected to be absent on mature RSV virion particles. This was partially explained by an early study, demonstrating that the presence of p27 peptide has a destabilizing effect on trimer formation and incorporation into virions (Krarup *et al*, 2015). However, some immature (unprocessed F0) molecules may be found at low frequency on virions (Krzyzaniak *et al*, 2013), explaining the weak binding of anti‐p27 serum to virions in ELISA.

In native F protein, the p27 contains 2 out of five glycosylation sites found in the F protein (N116 and N126). However, Leemans *et al* (2019) reported that infection of mice with recombinant virus lacking the N116 glycosylation site resulted in significantly higher neutralizing antibodies compared to wild‐type RSV infection expressing fully glycosylated RSV‐F. This finding further supports the hypothesis that fully glycosylated p27 is destabilizing the F trimer or interfere with proper folding of the F. In our study, the p27 peptide was unglycosylated (as chemically synthesized) and therefore could be more immunogenic than a fully glycosylated p27. Moreover, the antibodies elicited in mice or rabbits against p27 peptide stained both RSV‐infected cells *in vitro* and lung tissues from RSV challenged mice, suggesting that unglycosylated p27 peptide induced antibodies can recognize fully glycosylated p27 on RSV‐infected cells.

*In vitro* neutralization titers were high in the positive control animals (ID50 titers ranging between 1 × 10^3^ and 1 × 10^5^). Following F‐peptide immunization, neutralizing antibody responses were detected in animals vaccinated with peptides aa 147–203 (overlapping partial site Ø), aa 216–244, aa 234–287 (containing Site II), and aa 310–368 [site I]. One of these peptides (aa 234–287) includes site II, which is targeted by the neutralizing MAb palivizumab (Johnson *et al*, 1997). However, minimal neutralization of RSV was measured in the RSV‐LINT assay with sera from animals vaccinated with the p27 peptide. Therefore, the previously reported immunogenicity results with pre‐fusogenic F RSV vaccine candidate containing p27 (Patel *et al*, 2019), showing higher neutralizing titers are unlikely due to anti‐p27 antibodies contributing to the virus neutralization *in vitro*. The higher neutralizing activity of pre‐fusogenic F RSV vaccine candidate is potentially due to other neutralizing sites within F as was demonstrated by generation of higher titer antibodies by pre‐fusogenic F that competed with MAbs to site Ø, II, IV, and VIII compared with pre‐fusion or post‐fusion form of F (Patel *et al*, 2019). The study by Patel *et al* also suggests that inclusion of p27 in a vaccine may not negatively impact development of antibodies away from neutralization sites (sites Ø ‐IV) of RSV‐F.

It is formally possible that the strong p27 immunodominant response observed in infants after primary RSV infection could have evolved as an immune decoy mechanism to draw development of antibodies away from more effective epitopes in sites Ø ‐IV that are the principal neutralization sites on RSV‐F. To address this hypothesis, we challenged all vaccinated animals and evaluated their viral loads and lung pathology.

On day 5 following RSV challenge, significant control of lung viral loads was observed in the positive control groups (vaccinated with live A2 virus, pre‐ and post‐fusion F), and in animals vaccinated with several F peptides: aa 101–121 or 110–136 (spanning p27), aa 147–203 (partial site Ø), aa 216–244, and aa 234–287 (site II). These findings identified a discordance between the *in vitro* assays (Fig 2) and the *in vivo* protective activity of peptides containing p27 sequence (aa 101–121 or 110–136). Importantly, no increase in lung pathology scores was seen for most vaccinated/challenged animals compared with the PBS or KLH control groups at this antigen dose level (Fig 3). The lung histopathology scores following RSV challenge were similar among animals vaccinated with F peptides aa 101–121, aa 110–136 (p27), aa 443–461, pre‐F, and post‐fusion F proteins and the group immunized with RSV intranasally. Therefore, no evidence of enhanced respiratory disease was observed at the current antigen dose in the current study.

This study suggests that *in vivo* control of viral replication in RSV‐infected animals may be manifested by multiple mechanisms such as ADCC, cell‐mediated immunity (CMI), and Fc‐mediated binding and killing of RSV‐infected cells and immature virions expressing unprocessed F0, containing the p27 sequence, in addition to neutralization of cell free virions that express primarily mature F trimers. Cell surface staining of greater than 60% RSV‐infected A549 cells and lung tissues post‐RSV infection confirmed strong p27 cell surface expression (Figs 4 and 5). This suggests that a substantial number of RSV‐infected cells express F0 in the immature form containing p27 *in vivo* on their surface, in agreement with the study by Krzyzaniak *et al* (2013). Such cells could be targeted by anti‐p27 antibodies and subjected to killing via ADCC. We demonstrated that anti‐p27 antibodies mediate ADCC activity. Infected cells can also be targeted by cytotoxic T cells specific for p27‐derived short peptides loaded on MHC molecules. We observed increase in the number of both CD4 and CD8 cells in lung tissues from RSV‐infected (but not uninfected) mice. The numbers of T cells trended higher for vaccinated vs. PBS or KLH controls; however, they did not reach statistical significance and were not particularly elevated in the p27 vaccinated animals compared with the other F‐peptide vaccinated animals.

Together, these findings may explain the observed control of viral load and reduced lung pathology following challenge with RSV‐A2 virus in animals immunized with F‐p27 peptide. The findings in the previous and current study explain the robust anti‐p27 antibody responses observed in young children post‐primary RSV infection and suggest a functional protective role (rather than a decoy) of anti‐p27 immune response against RSV disease. F‐p27 demonstrates relatively high conservation (81–91%) among RSV‐A2 strains (Appendix Table S1, Appendix Fig S1). This is the first study that demonstrates F‐p27 as a protective target and provides proof‐of‐concept for the role of anti‐F‐p27 responses in protective immunity against RSV disease. Therefore, p27 could be added as a component of combinatorial vaccine approach for a better more effective vaccine against RSV.

## Materials and Methods

### Cell, viruses, and plasmids

A549 cells (Cat. No. #CCL‐185) were obtained from the American Type Culture Collection (ATCC, Manassas, VA, USA) and were maintained in F‐12K medium supplemented with 10% fetal bovine serum, 1X penicillin‐streptomycin (P‐S), and l‐glutamine. Cells were maintained in an incubator at 37°C under 5% CO_2_. RSV‐A2 strain (NR‐12149) was obtained from BEI resources, and viral stock (6.5 × 10^6^ pfu/ml) was generated using A549 cells. RSV rA2‐Line19F‐Firefly Luciferase (rRSV‐A2‐L19‐FFL), which expresses the firefly luciferase gene upstream of the NS1 gene, was prepared by infecting sub‐confluent A549 cell monolayers in F‐12K medium supplemented with 2% FBS and 1X penicillin‐streptomycin (infection medium) (Fuentes *et al*, 2017). To generate a challenge virus stock, at 5 days post‐infection (dpi), cells were freeze‐thawed twice, and virus was collected. Harvested viruses were cleared of cell debris by centrifugation at 3,795 *g* for 15 min. Virus stocks used in challenge studies were pelleted by centrifugation at 10,509 *g* overnight. Pelleted virus was resuspended in TNE buffer and purified by sucrose‐gradient ultracentrifugation (Fuentes *et al*, 2017). Virus titers were determined by plaque assay on A549 cells. The optimal challenge dose (10^6^ PFU/10 μl intranasally) was determined in an earlier study in which viral loads were measured by traditional plaque assay, by RT–qPCR, and by live imaging (flux) and gave comparable results in terms of viral kinetics and peak values (Fuentes *et al*, 2017).

### RSV‐F proteins and peptides

The genetically stabilized pre‐fusion RSV‐F (DS‐Cav1) plasmid encoding amino acid residues 26‐513Δ110‐136 of RSV‐A2 strain was a kind gift from Peter Kwong (VRC, NIH). DS‐Cav1 protein was produced in 293F cells (McLellan *et al*, 2013). The pre‐fusion conformation of the DS‐Cav1 protein was confirmed by binding to D25 MAb. Post‐fusion RSV‐F protein encodes amino acid residues 22–529Δ110–136 of RSV‐A2 strain fused to a polyhistidine tag produced in insect cells with endotoxin levels < 1 EU/µg of protein and was obtained from Sino Biologicals. The ‘wild‐type’ F protein showed very weak (> 100‐fold lower) binding to D25 MAb and primarily existed in a post‐fusion conformation. The RSV‐F peptides were chemically synthesized, purified by HPLC, conjugated to Keyhole limpet hemocyanin (KLH), and were used for animal vaccination as outlined below.

### Murine immunization, RSV challenge, and sample collection

Four‐ to 6‐week‐old female BALB/c mice (BALB/cAnNCr strain code #555) were obtained from Charles River Labs. Mice [*n* = 5 per group] were immunized intramuscularly (i.m.) at day 0 and day 28 with 20 μg of purified F protein or with 20 μg of KLH‐conjugated peptides combined with Emulsigen adjuvant, or with PBS (no vaccination control), or with 100 μg of unconjugated KLH alone (carrier control). As live RSV‐A2 virus control, a group of mice were given one intranasal dose of 10^4^ pfu/10 μl virus. Blood was collected from the tail vein on days 0 and 35. On day 42, mice were anesthetized with ketamine and dexmedetomidine cocktail given intraperitoneally according to mouse body weight and infected intranasally (i.n.) with 10^6^ PFU of rRSV‐A2‐L19‐FFL as previously described (Fuentes *et al*, 2017). Mice were sacrificed by CO_2_ asphyxiation 5 days post‐RSV challenge (previously determined to be the day with peak viral load), and blood and lungs were collected. For determination of the viral load analysis, the left lobe of the lung was collected; the right lung was used for histopathology analysis.

### RSV virion ELISA

Immulon 2 HB 96‐well microtiter plates were coated with 100 μl of purified RSV rA2‐Line19F‐FFL virus in PBS (10^4^ pfu/well) per well at 4°C overnight. After blocking with PBST containing 2% BSA, 5‐fold serial dilutions starting with an initial dilution of 1:100 mouse serum in blocking solution were added to each well, incubated for 1h at RT. This was followed by addition of 2,000‐fold dilution of HRP‐conjugated goat anti‐mouse IgG‐Fc specific antibody and developed by 100 µl of OPD substrate solution. Absorbance was measured at 490 nm. PBS immunized animals’ sera were used as a negative control, and a positive was defined as 2‐time greater absorbance than the negative control at each dilution.

### RSV‐luciferase inhibition neutralization test (RSV‐LINT)

A549 cells were seeded at 1.6 × 10^4^ cells in 100 µl of 10% FBS/1X penicillin‐streptomycin/F‐12K medium per well in 96‐well plates. Sera were heat inactivated for 30 min at 56°C. Ten‐fold diluted sera were further serially diluted 2‐fold from 1:10 to 1:2,560, added to RSV‐A2‐Rluc virus (final serum dilution of 1:20 to 1:5,120), and incubated at 37°C for 1 h. The luciferase assay was performed as described previously (Fuentes *et al*, 2013). Each serum dilution was run in duplicate, with appropriate positive and negative controls.

Luminescent readings for test samples are normalized using the virus only (no sera added) control values and multiplied by 100 to obtain percent of control. The percent of control values are used for linear regression analyses using the log_10_ of the reciprocal of the serum dilution on the X‐axis. The trendline of the linear part of the curve is used to calculate the slope and y‐intercept and 50% inhibition endpoint titers calculated using the formula: antilog of [(50 +y‐intercept)/slope]. If no neutralization was detected, a titer of 1 was assigned to samples.

### Determination of viral loads in lungs by RSV immuno‐plaque assay

Respiratory syncytial virus titers were measured in individual lung tissues at 5 days post‐RSV infection. Lungs (unperfused) were weighed and homogenized in DMEM, 7.5% BSA (5 ml medium/g of lung) using an Omni tissue homogenizer. The supernatant was cleared by centrifugation at 3,795 *g* for 10 min and used immediately for viral titration by plaque assay in HEp‐2 cells (human epidermoid carcinoma of larynx or HeLa‐derived epithelial cells, ATCC CCL‐23; from American Type Culture Collection, Manassas, VA). RSV plaques were determined using Immuno‐plaque assay (IPA) (Lee *et al*, 2018). HEp‐2 cells were grown in 48‐well plates until 90% confluent. Lung homogenates were serially diluted 5‐fold in DMEM without FBS. The lung samples were added to the plates and washed after incubation for 2 h at 37°C. Each well was overlaid with incomplete DMEM containing 0.8% low melting agarose and incubated for 4–5 days at 37°C. Cells were fixed with 4% neutral paraformaldehyde, and the agar plugs were removed. After washing the cells with 1% PBST, 1,000‐fold dilution of anti‐F palivizumab (Synagis) and then 1,000‐fold dilution of HRP‐conjugated anti‐human IgG (Fc) antibodies were added. Individual plaques were developed using DAB substrate kit (Invitrogen) and counted using light microscopy. The viral loads are reported as PFU/gram of lung tissue.

### Lung histopathology

The lung histopathology was performed after euthanizing mice with anesthetic cocktail containing ketamine and dexmedetomidine. Lung tissues were collected at 5 days post‐infection and fixed with 10% neutral‐buffered‐formalin for 24 h, stained with hematoxylin and eosin (H&E), and analyzed under light microscopy. The tissue slides were examined for characteristics including epithelial alterations in alveolitis, bronchiolitis, perivascular, and interstitial space (Lee *et al*, 2018). Lung tissues from individual mice (8 fields examined per animal) were scored blindly for histopathology analysis. Inflammation and focal aggregates of infiltrating epithelial alveolar cells in the airways, blood vessels, and interstitial space were examined blindly, and measured using a severity‐score system defined as 0 (no lesion; normal), 1 (mild inflammation; hypertrophy of bronchiolar cells, < 20% of lung affected), 2 (moderate inflammation; normal thickness of a single cuboidal cell, 20–40% of lung affected), 3 (marked inflammation; slight expansion and distension of the airway, 40–60% lung affected), and 4 (severe inflammation, thickening that occludes the airway lumen resulting in a very narrow lumen, > 60% lung affected with tissue necrosis or damage; Lee *et al*, 2018).

### RSV F‐p27 peptide ELISA

Immulon 2 HB 96‐well microtiter plates were coated with 100 μl of streptavidin in PBS (100 ng/well) at 4°C overnight. Biotinylated F‐p27 (110–136) peptide encompassing the entire p27 or peptide (aa 101–121) was captured, followed by blocking with PBST containing 2% BSA. Fivefold serial dilutions (starting dilution of 1:100 mouse serum) of post‐vaccination mouse serum in blocking solution were added to each well, incubated for 1 h at RT, followed by addition of 2,000‐fold dilution of HRP‐conjugated goat anti‐mouse IgG‐Fc‐specific antibody, and developed by 100 µl of OPD substrate solution. Absorbance was measured at 490 nm. PBS immunized animal serum was used as a negative control.

### Immunofluorescence assay (IFA) for p27 staining of RSV‐infected cell surface

A549 cells (ATCC CCL‐185; from American Type Culture Collection, Manassas, VA) were cultured in Nunc™ Lab‐Tek™ II Chambered Cover glass. When the confluency reaching 70–80%, cells were infected with RSV at a MOI of 0.1. At 16 h. post‐infection, cells were fixed with 4% paraformaldehyde for 30 min. Since we wanted to evaluate the presence of p27 on the cell surface, no cell permeabilization was performed. After three washes with PBST, 5% bovine serum albumin (BSA) containing PBST was added for 1 h, followed by incubation individually with 200‐fold dilution of rabbit serum generated against RSV‐F or F‐p27 (110‐136 residues), overnight at 4°C. Cells were then processed with 1,000‐fold dilution of anti‐rabbit Alexa Fluor 594 secondary antibody (Catalog number: 111‐545‐045; Jackson ImmunoResearch) for 1 h followed by 4′,6‐diamidino‐2‐phenylindole‐dihydrochloride (DAPI) for 5 min at room temperature. After three washes with PBST, cells were examined with a Leica SP8 confocal microscope. Percentage of cells was calculated by number of cells detected positive by anti‐RSV‐F or F‐p27 serum antibodies per at least 200 DAPI‐positive cells.

### Immunohistochemistry (IHC) for p27 staining of mouse lungs

Whole‐lung tissues were fixed in paraformaldehyde (PFA) and embedded in paraffin and used for slides. Four‐micrometer‐thick tissue sections were deparaffinized and boiled in antigen retrieval buffer (10 mM trisodium citrate, 0.05% Tween‐20) for 30 min. Slides were incubated overnight at 4°C with the 200‐fold dilution of primary antibodies (rabbit anti‐RSV‐F, or anti‐F‐p27 residues 110–136, or control serum) in a humidified chamber, followed by incubation with 1,000‐fold dilution of Alexa flour‐labeled anti‐rabbit secondary antibody (Catalog number: 111‐545‐045; Jackson ImmunoResearch). Nuclei were stained and mounted with ProLong™ Diamond Antifade Mountant with DAPI (Invitrogen, Camarillo, CA). Tissues were visualized using a Leica SP8 confocal microscope.

### Immunohistochemistry for CD4/CD8 T‐cell staining

Right lung tissues were harvested on day 5 post‐RSV infection and fixed with 10% neutral‐buffered formalin. They were embedded in paraffin and sections of tissue blocks were obtained. Four‐micrometer‐thick tissue sections were deparaffinized and boiled in antigen retrieval buffer (10 mM trisodium citrate, 0.05% Tween‐20) for 30 min, followed by blocking with 10% normal goat serum in PBS at room temperature for 60 min. Slides were incubated overnight at 4°C with the 200‐fold dilution of primary antibodies (rabbit anti‐CD4; ab183685 and rat anti‐CD8 alpha antibody; ab22378 from Abcam) in a humidified chamber. The slides followed by incubation with 1,000‐fold dilution of Alexa Fluor 488‐labeled AffiniPure Goat Anti‐Rabbit IgG (H + L); 111‐545‐045 or Alexa Fluor‐594‐labeled AffiniPure Goat Anti‐Rat IgG (H + L); 112‐585‐062 (Jackson ImmunoResearch). Nuclei were stained and mounted with ProLong™ Diamond Antifade Mountant with DAPI (Invitrogen, Camarillo, CA). Tissues were visualized using a Leica SP8 confocal microscope.

### Antibody‐dependent cellular cytotoxicity (ADCC) assay

Antibody‐dependent cellular cytotoxicity activity was assessed with a Promega ADCC Reporter Bioassay (Murine FcγRIV assay; M1201; Promega). Target cells (A549, either mock transfected or infected with 0.3 MOI of RSV‐A2 virus) for 24 h at 37°C, 5% CO_2_. After RSV or mock infection, cells were washed with PBS and cells were resuspended with RPMI with low IgG serum at a final concentration of 4 × 10^5^ cells/ml. The target cells were aliquoted at 25 μl per well into the assay plate. Post‐second immunization serum at a final dilution of 1:5 was added to each experimental well and allowed to incubate at room temperature for 30 min, while the effector cells were prepared. Genetically engineered Jurkat T‐cell line that expresses mouse FcγRIV along with a luciferase reporter driven by an NFAT‐response element (NFAT‐RE) as ADCC effector cells were added at the E:T ratio of 8:1 to the experimental wells. The plates are then incubated at 37°C, 5% CO_2_ for 6 h before detection. The activation of effector cells was detected with luciferase activity. RSV‐specific ADCC induction activity is calculated as the fold‐change of serum luciferase activity (RLU) of effector cells stimulation from RSV‐infected target cells compared with that from uninfected mock target cells. All data are normalized with plate background and no antibody control background before calculation of ADCC induction. All samples were tested in duplicate, and data shown are the mean of two biologically independent experiments.

### Ethics statement

All animal experiments were approved by the U.S. FDA Institutional Animal Care and Use Committee (IACUC) under Protocol #2009‐20. The animal care and use protocol meet National Institutes of Health (NIH) guidelines and complied with all ethical regulations.

### Statistical analysis

Five female BALB/c mice aged 4–6 weeks per group were used in the study. Power analysis calculations were done assuming a power value (beta) as 0.95, 0.9, and 0.8, in the order of decreasing stringency to eliminate Type I error. A significance level of 0.05 was used for sample size calculations. These calculations showed that we needed a sample size of 5.4, 4.3, and 3.1, respectively, that are within the actual sample size (*n* = 5) used in the current study. No data were excluded. There were no exclusion criteria. All samples and data were used for analysis and presented in the study. Animals were allocated randomly to each test group. Mice were allocated to different groups in equal sample size number (*n* = 5) and in a randomized fashion to eliminated selection bias. Experiments and data analysis were performed by different investigators, who were blinded to sample identity. The lung tissue slides were analyzed for image collection (confocal microscopy), image analysis, and scoring in an allocation concealing and blinded fashion (researcher was blinded to sample identity) to minimize selection bias and detection bias, respectively. The statistical significances of group differences were determined using one‐way analysis of variance (ANOVA) in GraphPad Prism, which assumes normal distribution of population, sample independence, and equality of variance. Correlations were calculated with a Spearman two‐tailed test. *P* values < 0.05 were considered significant with a 95% confidence interval.

## Author contributions

SK designed research. JL, LK, YL, EMC, SR, JT, and SK performed research. SK and HG contributed to writing.

## Conflict of interest

The authors declare that they have no conflict of interest.

## Supporting information



Appendix

Source Data for Figure 2

Source Data for Figure 3

Source Data for Figure 4

Source Data for Figure 6

## Data Availability

This study includes no data deposited in external repositories. All individual data are provided in the figures and supplementary information.
