# Supplementary material for: Protective antigenic sites identified in respiratory syncytial virus fusion protein reveals importance of p27 domain
Source: EMBO Mol Med. 2021 Nov 8;14(1):e13847. doi: 10.15252/emmm.202013847 (PMC8749483; doi:10.15252/emmm.202013847)
Supplement: Supplementary file 1 — Appendix [file EMMM-14-e13847-s006.pdf]

APPENDIX

Appendix to: Jeehyun Lee, Youri Lee, Laura Klenow, Elizabeth M Coyle, Juanjie Tang, Supriya Ravichandran, Hana Golding, and Surender Khurana. *PROTECTIVE ANTIGENIC SITES IDENTIFIED IN RESPIRATORY SYNCYTIAL VIRUS FUSION PROTEIN REVEALS IMPORTANCE OF p27 DOMAIN*

TABLE OF CONTENTS

1. Appendix Table S1 **Page 2**

2. Appendix Figure S1 **Page 3**

Appendix Table S1: Sequence Identity (%) of F antigenic site sequences among RSV strains

Sequence	A2	19F	2-20	Riyadh	A1998-12-21	Long	RSV-12	Bernett	Memphis-37	ON67-1210A	B1	B-CH18537
F	100	97.2	97.2	97.2	96.6	98	97.5	98.4	96.8	96.1	89.1	89
F 1-34	100	91.4	85.7	85.7	85.7	91.4	85.7	91.4	85.7	85.7	54.2	54.2
F 23-74	100	96.1	96.1	98	98	98	98	98	98	98	90.3	90.3
F 101-121	100	85.7	90.4	80.9	85.7	85.7	85.7	90.4	85.7	80.9	61.9	61.9
F 147-203	100	96.4	98.2	98.2	98.2	98.2	98.2	98.2	96.4	98.2	92.9	91.2
F 216-244	100	100	100	100	100	100	100	100	100	100	93.1	93.1
F 234-287	100	100	100	98.1	100	100	100	100	100	98.1	96.2	96.2
F 310-358	100	97.9	100	100	100	100	100	100	100	100	95.8	95.8
F 341-358	100	94.4	100	100	100	100	100	100	100	100	94.4	94.4
F 371-400	100	96.6	93.3	93.3	93.3	96.6	93.3	96.6	93.3	93.3	86.6	86.6
F 425-450	100	96.1	96.1	96.1	96.1	96.1	96.1	96.1	96.1	96.1	96.1	96.1
F 443-461	100	94.7	94.7	94.7	94.7	94.7	94.7	94.7	94.7	94.7	89.4	89.4
F 471-493	100	100	100	100	100	100	100	100	100	100	95.6	95.6
F 497-521	100	100	100	100	96	96	100	100	96	100	92	92
F 552-572	100	95.2	100	100	100	100	100	100	100	85.7	90.4	90.4

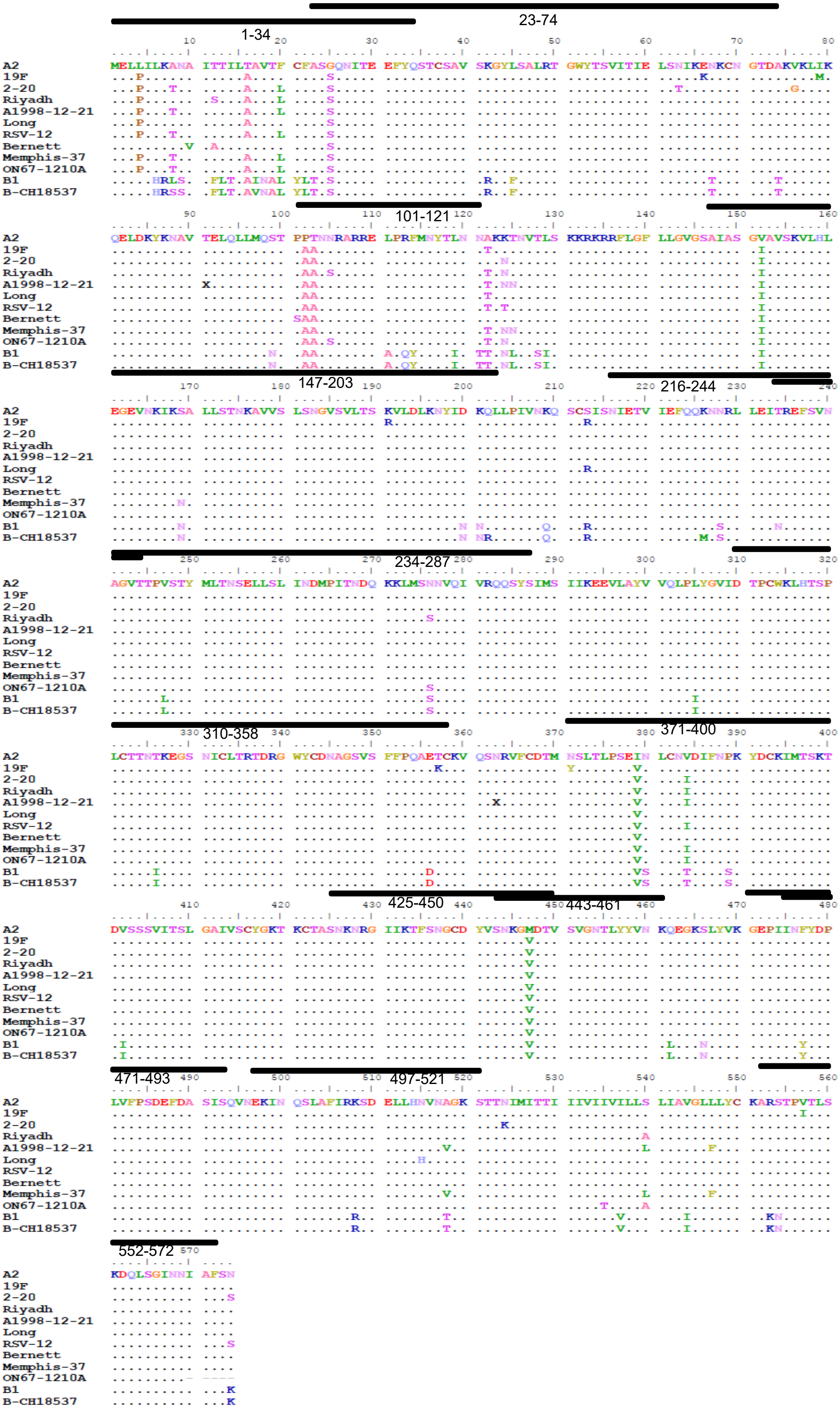

Appendix Figure S1: Sequence alignment of RSV F from diverse RSV strains. RSV strains include A2, A2001-2-20, A/Riyadh/2009, A1998-12-21, Long, RSV-12, A-Bernett-61, Memphis-37, ON67-1210A, B1, and B-CH18537. RSV F peptides that were used to immunize mice are displayed in black lines.
