# Supplementary material for: Protective antigenic sites identified in respiratory syncytial virus fusion protein reveals importance of p27 domain
Source: EMBO Mol Med. 2021 Nov 8;14(1):e13847. doi: 10.15252/emmm.202013847 (PMC8749483; doi:10.15252/emmm.202013847)
Supplement: Supplementary file 6 [file EMMM-14-e13847-s001.pdf]

EMBO Molecular Medicine

PROTECTIVE ANTIGENIC SITES IDENTIFIED IN RESPIRATORY SYNCYTIAL VIRUS FUSION PROTEIN REVEALS IMPORTANCE OF p27 DOMAIN

Jeehyun Lee, Youri Lee, Laura Klenow, Elizabeth Coyle, Juanjie Tang, Supriya Ravichandran, Hana Golding, and Surender Khurana
DOI: 10.15252/emmm.202013847

Corresponding authors: Surender Khurana (Surender.Khurana@fda.hhs.gov)

Review Timeline:

Submission Date:	16th Dec 20
Editorial Decision:	1st Feb 21
Revision Received:	25th Aug 21
Editorial Decision:	14th Oct 21
Revision Received:	22nd Oct 21
Accepted:	26th Oct 21

Editor: Zeljko Durdevic

Transaction Report:

(Note: With the exception of the correction of typographical or spelling errors that could be a source of ambiguity, letters and reports are not edited. Depending on transfer agreements, referee reports obtained elsewhere may or may not be included in this compilation. Referee reports are anonymous unless the Referee chooses to sign their reports.)

1st Feb 2021

Dear Dr. Khurana,

Thank you for the submission of your manuscript to EMBO Molecular Medicine. We have now received feedback from the three reviewers who agreed to evaluate your manuscript. As you will see from the reports below, while referee #1 does not support publication of your work, referees #2 and #3 are more positive but also raise serious concerns that should be addressed in a major revision.

Addressing the reviewers' concerns in full will be necessary for further considering the manuscript in our journal, and acceptance of the manuscript will entail a second round of review. EMBO Molecular Medicine encourages a single round of revision only and therefore, acceptance or rejection of the manuscript will depend on the completeness of your responses included in the next, final version of the manuscript. For this reason, and to save you from any frustrations in the end, I would strongly advise against returning an incomplete revision.

We realize that the current situation is exceptional on the account of the COVID-19/SARS-CoV-2 pandemic. Therefore, please let us know if you need more than six months to revise the manuscript.

I look forward to receiving your revised manuscript.

Yours sincerely,

Zeljko Durdevic

Editor
EMBO

***** Reviewer's comments *****

Referee #1 (Comments on Novelty/Model System for Author):

1)In the design of this paper, KLH was used as the peptide carrier, but there was a lack of comparison of the carrier itself, however the vector effect was important in the evaluation of immunogenicity and inflammation.

Referee #1 (Remarks for Author):

Attempts to develop an RSV vaccine over the past 60 years have spawned corresponding applications of new tools to discern the critical attributes that are needed for an effificacious vaccine. The surface exposed RSV fusion (F) glycoprotein is required for membrane fusion and infection and is a desirable vaccine candidate. Identification of protective epitopes in RSV proteins are critical for the development of effective vaccine against RSV disease. This manuscript identified an F peptide mapping within the p27 domain as a protective immunogen in vivo, which showed significant control of viral loads with significantly reduced pathology in the RSV-challenged lungs compared with mock-vaccinated controls, suggesting that F-p27 may should be included in an effective RSV vaccine. However, I regret to inform you that I have to reject this paper because of the following problems:

1)The advantages of including P27 epitopes to vaccine design were not fully demonstrated, The results of this paper show that p27 peptide plays a role through mechanisms such as ADCP instead of neutralizing. However, further research evidence is lacking, as we know that the Prefusogenic F vaccine, designed by Gale Smith et al., contains a p27 peptide and effectively increases the serum neutralization titer after immunization, what do you think of the difference in results?
2)In addition, although the expression of F0 can indeed be detected on the surface of infected cells and lung tissue, the positive

rate of F0 does not seem to be high according to the immunofluorescence results. Could the percentage of positive cells be provided to further support this conclusion? If the positive rate of these cells is not high, does adding P27 to vaccine design really have an advantage? Langedijk JPM et al. reported that the existence of P27 would lead to the destablization of the F trimer, which is likely to affect the identification of other epitopes on which the quaternary structure depends. Can the introduction of P27 make up for the loss of this component epitope?
3)In fact, Novavax had already announced that ResVaxTM(which incluing p27 peptide), RSV F vaccine for infants via maternal immunization, failed to meet the trial's primary efficacy endpoint in 2019, which is a heavy blow to RSV vaccine research. Please the authors share your constructive opinions for the failure of Novartis RSV vaccine.
4)In the design of this paper, KLH was used as the peptide carrier, but there was a lack of comparison of the carrier itself, however the vector effect was important in the evaluation of immunogenicity and inflammation.
5)The failure of the FI RSV is due in part to the fact that the vaccine does not activate effective neutralizing antibodies but activates binding antibodies, leading to the production of the final ERD. Peptides 1-34 and 23-74 both evoked higher levels of binding rather than neutralizing antibodies, but lower levels of inflammation. In contrast, the levels of binding antibodies stimulated by 147-203 were lower, and the levels of neutralizing antibodies were slightly higher, why the levels of inflammation were higher.

Referee #2 (Remarks for Author):

This is a follow-up study to a previous survey of neutralizing antibodies in infants, published by this group. In the present study submitted for publication in EMBO Molecular Medicine, mice were inoculated with individual RSV-F-derived peptides representing the antigenic sites previously identified in 66 RSV infected humans. This was followed by challenge of the inoculated mice with the RSV rRSV-A2-L19 strain. Of particular interest in this study was the vaccination of mice with peptides representing the p27 sequence in the RSV-F glycoprotein. The p27 sequence is cleaved from the precursor F0 protein that liberates the RSV fusion peptide in the RSV-F1 subunit. The p27 peptide therefore is not found on infectious RSV virions after peptide activation and is not represented as a neutralizing epitope on RSV. Immunodominant epitopes have been identified in RSV-Fp27 previously (PLoS Pathog 2016 Apr 21;12(4):e1005554) and in a handful of other papers. However they have not been studied further. The question remains as to how p27 peptides protect from RSV infection. This suggests the importance of cell-mediated immunity involving CD8 T cells. With a more elaborate hypothesis driven and mechanistic approach I think that this paper could represent a major advancement in our understanding of protection from RSV infection. Information derived from this work could lead to development of an effective RSV vaccine that has remained elusive for the better part of 70 years since the discovery of RSV.

I feel that more in vitro experiments need to be done to support their claims that an immune response to p27 is a productive response. As I outline below this includes ELISpot assays and some more imaging experiments of the distal lungs of the mice. The response that is directed by the p27 peptide needs to be characterized in other words. Furthermore, the paper appears to have been written in haste. There are many grammatical errors and word omissions that suggest this is an initial draft of a manuscript.

1. One must ask whether p27 antibodies are an important and therefore desirable aspect of protection from RSV infection. This is a drawback to the study that needs to be acknowledged and discussed in the discussion section at least. What I mean by this is that the strong p27 immunodominant response could have evolved as an immune decoy mechanism to draw development of antibodies away from more effective epitopes. That is, away from sites 0 - IV that are the principle neutralisation sites of RSV-F.

2. Another aspect that the authors have not considered is that RSV might not be proteolytically activated until it reaches the target cell. In other words, it may be the target cell and not the producer cell that express the protease necessary to activate the RSV virion before fusion and entry. This aspect should be considered by the authors and at least discussed.

3. The graph in Figure 2E is difficult to understand. The authors label the Y axis with IC50 titer but this is not how neutralizations are presented. If they are indicating IC50 then that is the concentration of antibody or chemical necessary to inhibit 50 % of infection. The neutralization titer is normally the reciprocal of the titration of antibody necessary to inhibit 50 % of infection. In other words, I suggest that the authors stick to a convention when presenting this data rather than mixing different methods of presenting inhibition data into one graph.

4. The data in figure 2E suggest that antibodies produced by inoculating with p27 peptides do not neutralize cell free viruses. This suggests that p27 peptides may induce cytotoxic mediated responses. Therefore I feel it is apt to suggest that the authors analyse the CD8 and CD4 T cell responses to these peptides in their system. For this they could analyse the T cell subsets with flow cytometry and ELISpot assays. This would likely yield important information on this aspect of RSV vaccine development and the immune response that would be valuable in vaccine development.

5. The authors have done a very good job of imaging mouse distal lungs. I suggest that more images of CD4 and CD8 cells could help visually support the possible roles of these cells in the immune response to RSV-Fp27. Are there more CD8 T cells with p27 peptide inoculation compared to inoculation by other peptides?

Minor criticisms. The data presented as a table in Figure 1 is unnecessary. The peptides used are useful but they could be included in a graphical representation of RSV-F polypeptide showing to what region in the RSV-F glycoprotein they correspond. This would be a more effective guide for the reader.

In the figure 2 legend they are missing the day post-RSV challenge. Refer to grammar and proofreading suggestion below.

Grammar.
Grammar needs significant attention. There are consistent instances where prepositions have been omitted. This makes the writing seem sloppy.

There are a number of split infinitives. For example, "We for the first time show"... should be written 'We show for the first time' or 'for the first time' we show. This will improve the quality of the writing. There is a repetitive use of the term, "for the first time." Use it once in the beginning of the paper such as the abstract and then once in the discussion.

Line 484. "Figure 4. Expression of p27 in the surface of RSV infected HEp-2 cells." This sentence should be correctly to 'on' the surface of RSV infected Hep-2 cells.

Referee #3 (Comments on Novelty/Model System for Author):

The model system, Balb/c mice are generally accepted for first evaluations of RSV infection biology.

Referee #3 (Remarks for Author):

Review: PROTECTIVE ANTIGENIC SITES IDENTIFIED IN RESPIRATORY SYNCYTIAL VIRUS FUSION PROTEIN REVEALS IMPORTANCE OF p27 DOMAIN

Remarks:
General:
- The manuscript describes the role of p27, a peptide of the RSV f protein which was previously considered not to be present on RSV virion, as a target for neutralization.
- This manuscript is well written, both grammatically and content-wise, and contains interesting results that can be very relevant for RSV vaccine development.
- The experiments that were performed are all relevant, correctly described and well executed.

Abstract: no remarks

Introduction:
- This introduction does not really represent the current state-of-art. The authors do not go into detail about the rationale for this study (this is however very briefly mentioned in 'the paper explained'), they mainly refer to their own previous work, which is nevertheless very relevant. Other publications could also be relevant, see also below.
- It seems odd to already summarize the most important results in the introduction.
- RSV has been renamed to human Orthopneumovirus (hOPV) a couple of years ago, and although RSV is still widely used as a name, it might be relevant to at least mention this.

Results:
- Line 79-93: This part is a repetition from what is written in the methods section and also the first part of the discussion. I would suggest avoiding these repetitions.
-

Discussion:
- Line 193: "ADCC, CMI". Abbreviations should be written in full when used for the first time.
- Line 203: "ADCP, CDP". Abbreviations should be written in full when used for the first time.
- The discussion is could be improved in my opinion and reference could be made to other publications. The authors do not go into depth about possible explanations of hypotheses for their findings. Why does a peptide that is believed to be cut out of the mature F protein elicit such a strong neutralizing antibody response? Similarly, if the p27 is still present in the F protein on the cell surface or in the F incorporated in virions, what would the consequences be for virus infectivity and the presence of other known neutralization sites? The p27 protein is heavily glycosylated with 3 N-glycans in this small peptide. A recent publication looked at the role of N-glycans in the p27 of RSV F and showed that removal of these glycosylations increase induction of neutralizing antibodies (Leemans et al., "Characterization of the role of N-glycosylation sites in the respiratory syncytial virus fusion protein in virus replication, syncytium formation and antigenicity". Virus Res. 2019 Jun;266:58-68."). Since N-glycans could be involved in 'glycan shielding) of the p27 peptide, this seems relevant to be discussed.

-

Methods:
- Line 225: I'm not sure if this freeze/thaw method is the most efficient method to prepare virus stocks, as this could result in a lot of debris in the virus preparation.
- Line 249: The authors do not mention immunization of mice with a negative control in the methods-section, however this is described in the results-section. This should be added in the methods-section to be complete.
- Line 254: When lung histopathology analysis is performed, anesthesia with isoflurane followed by CO_2 asphyxiation can be of influence on cell counts afterwards. This has already been described.
- Line 325: No permeabilization was performed with triton or other detergents. This might have been better for cytoplasmatic staining?

Figure Legends:
- Line 451: It is not clear what is meant by 'in vivo imaging'. This is also not described in the methods-section.
- Line 455: "...lung viral titers at day post-RSV challenge" Day is not specified.
- Line 485-486: Incorrect/unclear syntax

Reviewer's comments:

Referee #1 (Comments on Novelty/Model System for Author):

1)In the design of this paper, KLH was used as the peptide carrier, but there was a lack of comparison of the carrier itself, however the vector effect was important in the evaluation of immunogenicity and inflammation.

Response: As suggested, we have performed additional experiments with KLH alone as carrier control (comparable to the dose contained in the KLH-peptide conjugate vaccines in this study) and data has been added throughout the revised manuscript.

Lines 89-94: To that end, RSV-F peptides were chemically synthesized, purified by HPLC, conjugated to KLH, and used for animal vaccination. BALB/c mice (N = 5 per group) were immunized intramuscularly twice with 20 µg of RSV pre-fusion or post-fusion forms of F protein (positive controls), or with the F peptides-KLH conjugates mixed with Emulsigen adjuvant, or with PBS (no vaccination control), or with 100 µg of unconjugated KLH alone (carrier control), or with a single intranasal dose of 10^4 pfu live RSV A2 virus (Fig. 1A).

Referee #1 (Remarks for Author):

Attempts to develop an RSV vaccine over the past 60 years have spawned corresponding applications of new tools to discern the critical attributes that are needed for an effificacious vaccine. The surface exposed RSV fusion (F) glycoprotein is required for membrane fusion and infection and is a desirable vaccine candidate. Identification of protective epitopes in RSV proteins are critical for the development of effective vaccine against RSV disease. This manuscript identified an F peptide mapping within the p27 domain as a protective immunogen

in vivo, which showed significant control of viral loads with significantly reduced pathology in the RSV-challenged lungs compared with mock-vaccinated controls, suggesting that F-p27 may should be included in an effective RSV vaccine. However, I regret to inform you that I have to reject this paper because of the following problems:

1)The advantages of including P27 epitopes to vaccine design were not fully demonstrated, The results of this paper show that p27 peptide plays a role through mechanisms such as ADCP instead of neutralizing. However, further research evidence is lacking, as we know that the Prefusogenic F vaccine, designed by Gale Smith et al., contains a p27 peptide and effectively increases the serum neutralization titer after immunization, what do you think of the difference in results?

Response: To address reviewer's comment, we performed additional experiments to determine the number of CD4 and CD8 T-cells in lungs of vaccinated mice following RSV challenge as well as ADCC activity of p27-imunized serum samples. The data has been added throughout the revised manuscript. The data is shown in new figure 6.

Lines 175-201:

Role of cell mediated immunity and antibody Fc effector function in anti-p27 mediated protection from RSV disease

The observed reduction in lung viral titers and lung pathology after challenge of mice vaccinated with p27 suggested that mechanisms other than neutralizing antibodies may play a role in protection. To evaluate number of CD4 or CD8 T-cells in lungs following RSV-infection, we stained sections of lungs collected from mice at day 5 post-RSV challenge and determined CD4 and CD8 T cells in both airway and distal lung tissues. As shown in Fig. 6A, in all RSV challenged mice there was influx of both CD4 and CD8 T cells compared with uninfected control animals. When compared with the PBS (unvaccinated) or KLH controls, some vaccinated animals showed higher number of either CD4 or CD8 cells, but these differences did not reach statistical significance for the p27 vaccinated group.

Antibody dependent cell cytotoxicity (ADCC) may play a role in control of RSV-mediated pathology. Therefore, to determine if anti-p27 antibodies mediate ADCC, post-second immunization serum was assessed with a Promega ADCC Reporter Bioassay kit. A549 target cells were either mock treated or infected with RSV-A2 virus, and incubated with post-second immunization sera, followed by the addition of genetically engineered Jurkat T cells that expresses mouse FcγRIV along with a luciferase reporter driven by an NFAT-response element (NFAT-RE) for 6 hours. The RSV-specific ADCC activity induced by the serum antibodies was calculated against RSV-infected target cells compared with mock A549 cells as baseline. Sera from uninfected mice did not induce Jurkat cell activation above the baseline (Fig. 6B). The highest level of ADCC activity was observed with sera from mice immunized with live A2 virus prior to viral challenge. Importantly, sera from mice vaccinated with p27 peptide showed ADCC activity that was higher compared

with sera from mice vaccinated with pre-fusion F, which does not contain p27 sequence (Fig. 6B). Together, these studies suggest that p27 immunization induces non-neutralizing protection mechanisms against RSV that includes Fc-mediated killing of infected cells via ADCC and possibly T cell mediated effector functions.

Lines 275-284: Such cells could be targeted by anti-p27 antibodies and subjected to killing via ADCC. We demonstrated that anti p27 antibodies mediate ADCC activity. Infected cells can also be targeted by cytotoxic T cells specific for p27-derived short peptides loaded on MHC molecules. We observed increase in the number of both CD4 and CD8 cells in lung tissues from RSV-infected (but not uninfected) mice. The numbers of T cells trended higher for vaccinated vs. PBS or KLH controls, however, they did not reach statistical significance, and were not particularly elevated in the p27 vaccinated animals compared with the other F-peptide vaccinated animals.

Methods (lines 430-459):

Immunohistochemistry for CD4/CD8 T-cell staining
Right lung tissues were harvested on day 5 post-RSV infection and fixed with 10% neutral buffered formalin. They were embedded in paraffin and sections of tissue blocks were obtained. Four-micrometer-thick tissue sections were deparaffinized and boiled in antigen retrieval buffer (10 mM trisodium citrate, 0.05% Tween 20) for 30 min, followed by blocking with 10% normal goat serum in PBS at room temperature for 60 min. Slides were incubated overnight at 4°C with the primary antibodies (rabbit-anti-CD4 and rat-anti-CD8 from Abcam) in a humidified chamber. The slides followed by incubation with Alexa Fluor 488-labeled anti-rabbit or Alexa Fluor-594-labeled anti-rat secondary antibodies. Nuclei were stained and mounted with ProLong™ Diamond Antifade Mountant with DAPI (Invitrogen, Camarillo, CA).

Antibody-dependent cellular cytotoxicity (ADCC) assay
ADCC activity was assessed with a Promega ADCC Reporter Bioassay (Murine FcγRIV assay; M1201; Promega). Target cells (A549, either mock transfected or infected with 0.3 MOI of RSV-A2 virus) for 24 hours at 37°C, 5% CO2. After RSV or mock infection, cells were washed with PBS and cells were resuspended with RPMI with low IgG serum at a final concentration of 4X105 cells/ml. The target cells were aliquoted at 25 µL per well into the assay plate. Post-second immunization serum at a final dilution of 1:5 was added to each experimental well and allowed to incubate at room temperature for 30 minutes while the effector cells were prepared. Genetically engineered Jurkat T cell line that expresses mouse FcγRIV along with a luciferase reporter driven by an NFAT-response element (NFAT-RE) as ADCC effector cells were added at the E:T ratio of 8:1 to the experimental wells. The plates are then incubated at 37°C, 5% CO2 for 6 hours before detection. The activation of effector cells was detected with luciferase activity. RSV specific ADCC induction activity is

calculated as the fold-change of serum luciferase activity (RLU) of effector cell stimulation from RSV infected target cells compared with that from uninfected mock target cells. All data are normalized with plate background and no antibody control background before calculation of ADCC induction. All samples were tested in duplicate, and data shown is the mean of the replicates.

The previously reported immunogenicity results with pre-fusogenic F RSV vaccine candidate containing p27 (Patel et al., 2019) showing higher neutralizing titers are unlikely due to anti-p27 antibodies contributing to the virus neutralization *in vitro*. This study demonstrated the generation of antibodies that competed with monoclonal antibodies (MAbs) to site Ø, II, IV, and VIII, but no evidence was provided to demonstrate the contribution of anti-p27 antibodies to *in vitro* virus neutralization or *in vivo* protection against RSV A2 virus challenge The higher neutralizing activity of pre-fusogenic F RSV vaccine candidate is potentially due to other neutralizing sites within F as was demonstrated by generation of higher titer antibodies by pre-fusogenic F that competed with MAbs to site Ø, II, IV, and VIII compared with pre-fusion or post-fusion form of F (Patel et al., 2019). The study by Patel et al., also suggests that inclusion of p27 in a vaccine may not negatively impact development of antibodies away from neutralization sites (sites Ø -IV) of RSV-F.

We have discussed these previous observations in the revised manuscript:

Lines 65-71: One RSV vaccine candidate based on a prefusogenic form of F containing p27 generated higher neutralizing antibodies compared with prefusion and post-fusion F proteins (lacking p27), and protected animals from RSV challenge in mice (Patel, Massare et al., 2019). In addition, this study demonstrated the generation of antibodies that competed with monoclonal antibodies (MAbs) to site Ø, II, IV, and VIII, but no evidence was provided to demonstrate the contribution of anti-p27 antibodies to in vitro virus neutralization or in vivo protection against RSV A2 virus challenge (Patel et al., 2019).

Lines 242-252: However, minimal neutralization of RSV was measured in the RSV-LINT assay with sera from animals vaccinated with the p27 peptide. Therefore, the previously reported immunogenicity results with pre-fusogenic F RSV vaccine candidate containing p27 (Patel et al., 2019) showing higher neutralizing titers are unlikely due to anti-p27 antibodies contributing to the virus neutralization in vitro. The higher neutralizing activity of pre-fusogenic F RSV vaccine candidate is potentially due to other neutralizing sites within F as was demonstrated by generation of higher titer antibodies by pre-fusogenic F that competed with MAbs to site Ø, II, IV, and VIII compared with pre-fusion or post-fusion form of F (Patel et al., 2019). The study by Patel et al., also suggests that inclusion of p27 in a vaccine may not negatively impact

development of antibodies away from neutralization sites (sites Ø -IV) of RSV-F.

2)In addition, although the expression of F0 can indeed be detected on the surface of infected cells and lung tissue, the positive rate of F0 does not seem to be high according to the immunofluorescence results. Could the percentage of positive cells be provided to further support this conclusion? If the positive rate of these cells is not high, does adding P27 to vaccine design really have an advantage? Langedijk JPM et al. reported that the existence of P27 would lead to the destablization of the F trimer, which is likely to affect the identification of other epitopes on which the quaternary structure depends. Can the introduction of P27 make up for the loss of this component epitope?

Response: As per reviewer's suggestions, we performed additional experiments to determine the number RSV-F and F-p27 expressing cells following RSV infection *in-vitro* and RSV-infected lungs from mice. Both these studies showed a high percentage (>60%) of in RSV-infected cells expressing similar levels of RSV-F as well as F-p27 on cell surface (revised figure 4) as well as in the lung tissue of RSV challenged mice (revised figure 5). We have added the data in revised manuscript and discussed it further.

Lines 154-173:

F-p27 is expressed on the surface of RSV-infected cells and in the lungs of RSV-infected mice.

While p27 (residues 110-136) is not part of the mature F protein on virions, some immature or unprocessed F0 may be present on virions (Krzyzaniak, Zumstein et al., 2013) and might not be proteolytically activated until it reaches the target cells. On the other hand, p27 is part of newly translated F0 in RSV-infected cells. As can be seen in Fig. 4A, all mice vaccinated with the p27-containing peptide (110-136 residues) generated p27-peptide (110-136) antibody binding titers in ELISA but did not bind to F (1-34) peptide (negative control). Antisera from animals given "pre-F" or "post-F" proteins lacking the p27 sequence did not react to the p27 (110-136) peptide in ELISA (data not shown).

To determine if the p27 peptide is expressed on the surface of RSV-infected cells, we generated rabbit antiserum against F protein and against F peptide containing residues 110-136 of p27 sequence. Immunostaining performed on non-permeabilized RSV A2-infected A549 cells demonstrated a comparable strong cell-surface staining patterns using either anti-RSV F protein or anti-F-p27 peptide antisera. Both antisera-stained similar percentages of RSV-A2 infected A549 cells (64.5% and 63.89 %, respectively) (Fig. 4B).

Furthermore, we also observed strong p27 expression in sections of lung tissues from RSV A2-infected animals using rabbit serum generated against either RSV F protein (62.25%) or F-p27 (110-136) peptide (61.59%) (Fig. 5). These observations suggest that F-p27-containing immature F0 is widely

expressed on surface of RSV-infected cells in vitro and in RSV-infected lungs in vivo.

Lines 267-284:

This study suggests that *in vivo* control of viral replication in RSV-infected animals may be manifested by multiple mechanisms such as Antibody-dependent cellular cytotoxicity (ADCC), cell-mediated immunity (CMI) and Fc-mediated binding and killing of RSV infected cells and immature virions expressing unprocessed F0, containing the p27 sequence, in addition to neutralization of cell free virions that express primarily mature F trimers. Staining of greater than 60% RSV-infected A549 cells and lung tissues post-RSV infection confirmed strong p27 cell surface expression (Figs. 4, 5). This suggests that a substantial number of RSV-infected cells express F0 in the immature form containing p27 *in vivo* on their surface, in agreement with the study by Krzyzaniak et al (Krzyzaniak et al., 2013). Such cells could be targeted by anti-p27 antibodies and subjected to killing via ADCC. We demonstrated that anti p27 antibodies mediate ADCC activity. Infected cells can also be targeted by cytotoxic T cells specific for p27-derived short peptides loaded on MHC molecules. We observed increase in the number of both CD4 and CD8 cells in lung tissues from RSV-infected (but not uninfected) mice. The numbers of T cells trended higher for vaccinated vs. PBS or KLH controls, however, they did not reach statistical significance, and were not particularly elevated in the p27 vaccinated animals compared with the other F-peptide vaccinated animals.

Lines 218-236:

The low binding of anti-p27 peptides to virions is explained by the fact that p27 is uniquely found in uncleaved F0 which is normally excised during F protein maturation into F1/F2 complex and is expected to be absent on mature RSV virion particles. This was partially explained by an early study demonstrating that the presence of p27 peptide has a destabilizing effect on trimer formation and incorporation into virions (Krarup et al., 2015). However, some immature (unprocessed F0) molecules may be found at low frequency on virions (Krzyzaniak et al., 2013), explaining the weak binding of anti-p27 serum to virions in ELISA.

In native F protein the p27 contains 2 out of five glycosylation sites found in the F protein (N116 and N126). However, Leemans et al., reported that infection of mice with recombinant virus lacking the N116 glycosylation site resulted in significantly higher neutralizing antibodies compared to wild-type RSV infection expressing fully glycosylated RSV-F (Leemans, Boeren et al., 2019). This finding further supports the hypothesis that fully glycosylated p27 is destabilizing the F trimer or interfere with proper folding of the F. In our study, the p27 peptide was unglycosylated (as chemically synthesized) and therefore could be more immunogenic than a fully glycosylated p27. Moreover, the antibodies elicited in mice or rabbits against p27 peptide stained both RSV infected cells in vitro and lung tissues from RSV challenged

mice, suggesting that unglycosylated p27 peptide induced antibodies can recognize fully-glycosylated p27 on RSV infected cells.

3)In fact, Novavax had already announced that ResVaxTM(which incluing p27 peptide), RSV F vaccine for infants via maternal immunization, failed to meet the trial's primary efficacy endpoint in 2019, which is a heavy blow to RSV vaccine research. Please the authors share your constructive opinions for the failure of Novartis RSV vaccine.

Response: Since all the authors are employees of US FDA, a regulatory agency within US govt. responsible for review and approval of vaccines in US, we are not in a position to comment or provide opinions on an investigational vaccine.

4)In the design of this paper, KLH was used as the peptide carrier, but there was a lack of comparison of the carrier itself, however the vector effect was important in the evaluation of immunogenicity and inflammation.

Response: As per reviewer's suggestion, we performed additional experiments with KLH alone as carrier control (comparable to the dose contained in the peptide vaccines in this study) for evaluation of immunogenicity and inflammation. All the data has been added in various figures and throughout the revised manuscript.

Lines 89-94: To that end, RSV-F peptides were chemically synthesized, purified by HPLC, conjugated to KLH, and used for animal vaccination. BALB/c mice (N = 5 per group) were immunized intramuscularly twice with 20 µg of RSV pre-fusion or post-fusion forms of F protein (positive controls), or with the F peptides-KLH conjugates mixed with Emulsigen adjuvant, or with PBS (no vaccination control), or with 100 µg of unconjugated KLH alone (carrier control), or with a single intranasal dose of 104 pfu live RSV A2 virus (Fig. 1A).

Lines 328-332: Four- to 6-week-old female BALB/c mice (BALB/cAnNCr strain code #555) were obtained from Charles River Labs. Mice [N = 5 per group] were immunized intramuscularly (i.m.) at day 0 and day 28 with 20 µg of purified F protein or with 20 µg of KLH-conjugated peptides combined with Emulsigen adjuvant, or with PBS (no vaccination control), or with 100 µg of unconjugated KLH alone (carrier control).

5)The failure of the FI RSV is due in part to the fact that the vaccine does not activate effective neutralizing antibodies but activates binding antibodies, leading to the production of the final ERD. Peptides 1-34 and 23-74 both evoked higher levels of binding rather than neutralizing antibodies, but lower levels of inflammation. In contrast, the levels of binding antibodies stimulated by 147-203 were lower, and the levels of neutralizing antibodies were slightly higher, why the levels of inflammation

were higher.

Response: As suggested, we expanded on this observation further in the revised manuscript.

Lines 148-152: Interestingly, peptides 1-34 and 23-74 elicited binding antibodies that did not neutralize the virus in vitro (Fig. 1B). Yet the lung pathology scores for these groups was highly variable and did not reach statistical significance compared with other groups (Fig. 3B). All together, we did not find evidence for enhanced lung pathology following challenge in any of the vaccinated groups at this antigen dose.

Referee #2 (Remarks for Author):

This is a follow-up study to a previous survey of neutralizing antibodies in infants, published by this group. In the present study submitted for publication in EMBO Molecular Medicine, mice were inoculated with individual RSV-F-derived peptides representing the antigenic sites previously identified in 66 RSV infected humans. This was followed by challenge of the inoculated mice with the RSV rRSV-A2-L19 strain. Of particular interest in this study was the vaccination of mice with peptides representing the p27 sequence in the RSV-F glycoprotein. The p27 sequence is cleaved from the precursor F0 protein that liberates the RSV fusion peptide in the RSV-F1 subunit. The p27 peptide therefore is not found on infectious RSV virions after peptide activation and is not represented as a neutralizing epitope on RSV. Immunodominant epitopes have been identified in RSV-Fp27 previously (PLoS Pathog 2016 Apr 21;12(4):e1005554) and in a handful of other papers. However they have not been studied further. The question remains as to how p27 peptides protect from RSV infection. This suggests the importance of cell-mediated immunity involving CD8 T cells. With a more elaborate hypothesis driven and mechanistic approach I think that this paper could represent a major advancement in our understanding of protection from RSV infection. Information derived from this work could lead to development of an effective RSV vaccine that has remained elusive for the better part of 70 years since the discovery of RSV.

Response: We thank the reviewer for appreciating the importance of our study.

I feel that more in vitro experiments need to be done to support their claims that an immune response to p27 is a productive response. As I outline below this includes ELISpot assays and some more imaging experiments of the distal lungs of the mice. The response that is directed by the p27 peptide needs to be characterized in other words. Furthermore, the paper appears to have been written in haste. There are many grammatical errors and word omissions that suggest this is an initial draft of a manuscript.

Response: We have performed additional experiments to address the comment as detailed in responses below. The manuscript has been revised to remove all typographical and grammatical errors.

1. One must ask whether p27 antibodies are an important and therefore desirable aspect of protection from RSV infection. This is a drawback to the study that needs to be acknowledged and discussed in the discussion section at least. What I mean by this is that the strong p27 immunodominant response could have evolved as an immune decoy mechanism to draw development of antibodies away from more effective epitopes. That is, away from sites 0 - IV that are the principle neutralisation sites of RSV-F.

Response: Based on the previous study by Patel et al, its unlikely that p27 immune response negatively impact the immune response to principal neutralization sites within RSV-F. We have discussed this further in the revised manuscript.

Lines 65-71: One RSV vaccine candidate based on a prefusogenic form of F containing p27 generated higher neutralizing antibodies compared with prefusion and post-fusion F proteins (lacking p27), and protected animals from RSV challenge in mice (Patel, Massare et al., 2019). In addition, this study demonstrated the generation of antibodies that competed with monoclonal antibodies (MAbs) to site Ø, II, IV, and VIII, but no evidence was provided to demonstrate the contribution of anti-p27 antibodies to in vitro virus neutralization or in vivo protection against RSV A2 virus challenge (Patel et al., 2019).

Lines 242-256: However, minimal neutralization of RSV was measured in the RSV-LINT assay with sera from animals vaccinated with the p27 peptide. Therefore, the previously reported immunogenicity results with pre-fusogenic F RSV vaccine candidate containing p27 (Patel et al., 2019) showing higher neutralizing titers are unlikely due to anti-p27 antibodies contributing to the virus neutralization in vitro. The higher neutralizing activity of pre-fusogenic F RSV vaccine candidate is potentially due to other neutralizing sites within F as was demonstrated by generation of higher titer antibodies by pre-fusogenic F that competed with MAbs to site Ø, II, IV, and VIII compared with pre-fusion or post-fusion form of F (Patel et al., 2019). The study by Patel et al., also suggests that inclusion of p27 in a vaccine may not negatively impact development of antibodies away from neutralization sites (sites Ø -IV) of RSV-F.

It is formally possible that the strong p27 immunodominant response observed in infants after primary RSV infection could have evolved as an immune decoy mechanism to draw development of antibodies away from more effective epitopes in sites Ø-IV that are the principal neutralization sites on RSV-F. To address this hypothesis, we challenged all vaccinated animals and evaluated their viral loads and lung pathology.

Lines 287-290: The findings in the previous and current study explain the robust anti-p27 antibody responses observed in young children post-primary RSV infection and suggest a functional protective role (rather than a decoy) of anti-p27 immune response against RSV disease.

2. Another aspect that the authors have not considered is that RSV might not be proteolytically activated until it reaches the target cell. In other words, it may be the target cell and not the producer cell that express the protease necessary to activate the RSV virion before fusion and entry. This aspect should be considered by the authors and at least discussed.

Response: Absence of anti-p27 serum reactivity to virion particles and presence of high amount of p27 on cell surface of virally infected cells, suggest that RSV-target cells upon RSV infection express immature RSV-F0 on cell surface as detected by similar number of RSV-F and F-p27 positive cells. We have discussed it further.

Lines 155-168: While p27 (residues 110-136) is not part of the mature F protein on virions, some immature or unprocessed F0 may be present on virions (Krzyzaniak, Zumstein et al., 2013) and might not be proteolytically activated until it reaches the target cells. On the other hand, p27 is part of newly translated F0 in RSV-infected cells. As can be seen in Fig. 4A, all mice vaccinated with the p27-containing peptide (110-136 residues) generated p27-peptide (110-136) antibody binding titers in ELISA but did not bind to F (1-34) peptide (negative control). Antisera from animals given "pre-F" or "post-F" proteins lacking the p27 sequence did not react to the p27 (110-136) peptide in ELISA (data not shown).

To determine if the p27 peptide is expressed on the surface of RSV-infected cells, we generated rabbit antiserum against F protein and against F peptide containing residues 110-136 of p27 sequence. Immunostaining performed on non-permeabilized RSV A2-infected A549 cells demonstrated a comparable strong cell-surface staining patterns using either anti-RSV F protein or anti-F-p27 peptide antisera. Both antisera-stained similar percentages of RSV-A2 infected A549 cells (64.5% and 63.89 %, respectively) (Fig. 4B).

3. The graph in Figure 2E is difficult to understand. The authors label the Y axis with IC50 titer but this is not how neutralizations are presented. If they are indicating IC50 then that is the concentration of antibody or chemical necessary to inhibit 50 % of infection. The neutralization titer is normally the reciprocal of the titration of antibody necessary to inhibit 50 % of infection. In other words, I suggest that the authors stick to a convention when presenting this data rather than mixing different methods of presenting inhibition data into one graph.

Response: We agree, and it has been revised to ID50.

Lines 594-5: End-point serum dilution titer that resulted in 50% inhibition of RSV infection (ID50) in RSV-LINT assay is shown.

4. The data in figure 2E suggest that antibodies produced by inoculating with p27 peptides do not neutralize cell free viruses. This suggests that p27 peptides may induce cytotoxic mediated responses. Therefore I feel it is apt to suggest that the authors analyse the CD8 and CD4 T cell responses to these peptides in their system. For this they could analyse the T cell subsets with flow cytometry and ELISpot assays. This would likely yield important information on this aspect of RSV vaccine development and the immune response that would be valuable in vaccine development.

Response: We agree with the reviewer.

We have not stored any blood/PBMC following vaccination of mice and prior to RSV-challenge. Due to ongoing COVID-19 pandemic, with restrictions on non-COVID research and limitations of both animal facility and resources available, it was not possible to repeat the large animal experiments and obtain post-vaccination PBMCs.

However, to address reviewer's comment, we performed additional experiments to determine the number of CD4 and CD8 T-cells in the lungs by immuno-staining the lung tissues collected at day 5 following RSV-infection of vaccinated mice and data has been added throughout the revised manuscript. The data is shown in new figure 6.

Lines 175-201:
Role of cell mediated immunity and antibody Fc effector function in anti-p27 mediated protection from RSV disease

The observed reduction in lung viral titers and lung pathology after challenge of mice vaccinated with p27 suggested that mechanisms other than neutralizing antibodies may play a role in protection. To evaluate number of CD4 or CD8 T-cells in lungs following RSV-infection, we stained sections of lungs collected from mice at day 5 post-RSV challenge and determined CD4 and CD8 T cells in both airway and distal lung tissues. As shown in Fig. 6A, in all RSV challenged mice there was influx of both CD4 and CD8 T cells compared with uninfected control animals. When compared with the PBS (unvaccinated) or KLH controls, some vaccinated animals showed higher number of either CD4 or CD8 cells, but these differences did not reach statistical significance for the p27 vaccinated group.

Antibody dependent cell cytotoxicity (ADCC) may play a role in control of RSV-mediated pathology. Therefore, to determine if anti-p27 antibodies mediate ADCC, post-second immunization serum was assessed with a Promega ADCC Reporter Bioassay. A549 target cells were either mock treated

or infected with RSV-A2 virus, and incubated with post-second immunization sera, followed by the addition of genetically engineered Jurkat T cells that expresses mouse FcγRIV along with a luciferase reporter driven by an NFAT-response element (NFAT-RE) for 6 hours. The RSV-specific ADCC activity induced by the serum antibodies was calculated against RSV-infected target cells compared with mock A549 cells as baseline. Sera from uninfected mice did not induce Jurkat cell activation above the baseline (Fig. 6B). The highest level of ADCC activity was observed with sera from mice immunized with live A2 virus prior to viral challenge. Importantly, sera from mice vaccinated with p27 peptide showed ADCC activity that was higher compared with sera from mice vaccinated with pre-fusion F, which does not contain p27 sequence (Fig. 6B). Together, these studies suggest that p27 immunization induces non-neutralizing protection mechanisms against RSV that includes Fc-mediated killing of infected cells via ADCC and possibly T cell mediated effector functions.

Lines 268-284: This study suggests that *in vivo* control of viral replication in RSV-infected animals may be manifested by multiple mechanisms such as Antibody-dependent cellular cytotoxicity (ADCC), cell-mediated immunity (CMI) and Fc-mediated binding and killing of RSV infected cells and immature virions expressing unprocessed F0, containing the p27 sequence, in addition to neutralization of cell free virions that express primarily mature F trimers. Staining of greater than 60% RSV-infected A549 cells and lung tissues post-RSV infection confirmed strong p27 cell surface expression (Figs. 4, 5). This suggests that a substantial number of RSV-infected cells express F0 in the immature form containing p27 *in vivo* on their surface, in agreement with the study by Krzyzaniak et al (Krzyzaniak et al., 2013). Such cells could be targeted by anti-p27 antibodies and subjected to killing via ADCC. We demonstrated that anti p27 antibodies mediate ADCC activity. Infected cells can also be targeted by cytotoxic T cells specific for p27-derived short peptides loaded on MHC molecules. We observed increase in the number of both CD4 and CD8 cells in lung tissues from RSV-infected (but not uninfected) mice. The numbers of T cells trended higher for vaccinated vs. PBS or KLH controls, however, they did not reach statistical significance, and were not particularly elevated in the p27 vaccinated animals compared with the other F-peptide vaccinated animals.

Methods (lines 430-459):

Immunohistochemistry for CD4/CD8 T-cell staining

Right lung tissues were harvested on day 5 post-RSV infection and fixed with 10% neutral buffered formalin. They were embedded in paraffin and sections of tissue blocks were obtained. Four-micrometer-thick tissue sections were deparaffinized and boiled in antigen retrieval buffer (10 mM trisodium citrate, 0.05% Tween 20) for 30 min, followed by blocking with 10% normal goat

serum in PBS at room temperature for 60 min. Slides were incubated overnight at 4°C with the primary antibodies (rabbit-anti-CD4 and rat-anti-CD8 from Abcam) in a humidified chamber. The slides followed by incubation with Alexa Fluor 488-labeled anti-rabbit or Alexa Fluor-594-labeled anti-rat secondary antibodies. Nuclei were stained and mounted with ProLong™ Diamond Antifade Mountant with DAPI (Invitrogen, Camarillo, CA).

Antibody-dependent cellular cytotoxicity (ADCC) assay

ADCC activity was assessed with a Promega ADCC Reporter Bioassay (Murine FcγRIV assay; M1201; Promega). Target cells (A549, either mock transfected or infected with 0.3 MOI of RSV-A2 virus) for 24 hours at 37°C, 5% CO2. After RSV or mock infection, cells were washed with PBS and cells were resuspended with RPMI with low IgG serum at a final concentration of 4X105 cells/ml. The target cells were aliquoted at 25 μL per well into the assay plate. Post-second immunization serum at a final dilution of 1:5 was added to each experimental well and allowed to incubate at room temperature for 30 minutes while the effector cells were prepared. Genetically engineered Jurkat T cell line that expresses mouse FcγRIV along with a luciferase reporter driven by an NFAT-response element (NFAT-RE) as ADCC effector cells were added at the E:T ratio of 8:1 to the experimental wells. The plates are then incubated at 37°C, 5% CO2 for 6 hours before detection. The activation of effector cells was detected with luciferase activity. RSV specific ADCC induction activity is calculated as the fold-change of serum luciferase activity (RLU) of effector cells stimulation from RSV infected target cells compared with that from uninfected mock target cells. All data are normalized with plate background and no antibody control background before calculation of ADCC induction. All samples were tested in duplicate, and data shown is the mean of the two biologically independent experiments.

5. The authors have done a very good job of imaging mouse distal lungs. I suggest that more images of CD4 and CD8 cells could help visually support the possible roles of these cells in the immune response to RSV-Fp27. Are there more CD8 T cells with p27 peptide inoculation compared to inoculation by other peptides?

Response: As per reviewer suggestions, we immuno-characterized the number of CD4 and CD8 T-cells in the lung tissues collected at day 5 following RSV-infection of vaccinated mice and data has been added throughout the revised manuscript. The data is shown in new figure 6.

See response to comment 4 above.

Minor criticisms. The data presented as a table in Figure 1 is unnecessary. The peptides used are useful but they could be included in a graphical representation of RSV-F polypeptide showing to what region in the RSV-F glycoprotein they correspond. This would be a more effective guide for the reader.

Response: As per reviewer's suggestions we have added the schematic to graphically represent different peptides corresponding to RSV-F used in the study (new figure 1B). We feel that Table 1A captures the whole study design and therefore have retained it in figure 1.

In the figure 2 legend they are missing the day post-RSV challenge. Refer to grammar and proofreading suggestion below.

Response: Thanks for catching the error and it has been removed. The figure legend has been proof-read and corrected.

Grammar.
Grammar needs significant attention. There are consistent instances where prepositions have been omitted. This makes the writing seem sloppy.

Response: We have corrected all typographical errors and grammatical mistakes thought out the manuscript. The entire manuscript has been proof-read.

There are a number of split infinitives. For example, "We for the first time show"... should be written 'We show for the first time' or 'for the first time' we show. This will improve the quality of the writing. There is a repetitive use of the term, "for the first time." Use it once in the beginning of the paper such as the abstract and then once in the discussion.

Response: Thanks for pointing them out. All these issues have been fixed in the revised manuscript.

Line 484. "Figure 4. Expression of p27 in the surface of RSV infected HEp-2 cells." This sentence should be correctly to 'on' the surface of RSV infected Hep-2 cells.

Response: Thanks for catching the error. We repeated the experiment with A549 cells. It has been corrected to:

Figure 4. Expression of p27 on the surface of RSV infected A549 cells.

Referee #3 (Comments on Novelty/Model System for Author):

The model system, Balb/c mice are generally accepted for first evaluations of RSV infection biology.

Referee #3 (Remarks for Author):

Review: PROTECTIVE ANTIGENIC SITES IDENTIFIED IN RESPIRATORY SYNCYTIAL VIRUS FUSION PROTEIN REVEALS IMPORTANCE OF p27 DOMAIN

Remarks:
General:
- The manuscript describes the role of p27, a peptide of the RSV f protein which was previously considered not to be present on RSV virion, as a target for neutralization.
- This manuscript is well written, both grammatically and content-wise, and contains interesting results that can be very relevant for RSV vaccine development.
- The experiments that were performed are all relevant, correctly described and well executed.

Response: We thank the reviewer for appreciating our study.

Abstract: no remarks

Introduction:
- This introduction does not really represent the current state-of-art. The authors do not go into detail about the rationale for this study (this is however very briefly mentioned in 'the paper explained'), they mainly refer to their own previous work, which is nevertheless very relevant. Other publications could also be relevant, see also below.

Response: We have expanded the introduction to add information on previous prefusogenic F study containing p27 sequence in the immunogen.

Lines 65-71: One RSV vaccine candidate based on a prefusogenic form of F containing p27 generated higher neutralizing antibodies compared with prefusion and post-fusion F proteins (lacking p27), and protected animals from RSV challenge in mice (Patel, Massare et al., 2019). In addition, this study demonstrated the generation of antibodies that competed with monoclonal antibodies (MAbs) to site Ø, II, IV, and VIII, but no evidence was provided to demonstrate the contribution of anti-p27 antibodies to in vitro virus neutralization or in vivo protection against RSV A2 virus challenge (Patel et al., 2019). Moreover, there is limited information on antigenic sites within F that provide protection that are key for development of an effective RSV vaccine.

- It seems odd to already summarize the most important results in the introduction.

Response: We have removed the results summary from the introduction.

- RSV has been renamed to human Orthopneumovirus (hOPV) a couple of years

ago, and although RSV is still widely used as a name, it might be relevant to at least mention this.

Response: Thanks. We have added this information.

Lines 47-50: Significant efforts are underway to develop and evaluate RSV vaccines targeted to pregnant women with hope of protecting neonates from RSV [renamed to human Orthopneumovirus (hOPV)] induced lung disease early in life, as well as to elderly populations, who are susceptible to recurrent RSV infections (Drysdale, Barr et al., 2020).

Results:
- Line 79-93: This part is a repetition from what is written in the methods section and also the first part of the discussion. I would suggest avoiding these repetitions.

Response: We have revised the section to remove any repetition.

Discussion:
- Line 193: "ADCC, CMI". Abbreviations should be written in full when used for the first time.

Response: We have provided the full form of abbreviations at their first occurrence in the manuscript.

- Line 203: "ADCP, CDP". Abbreviations should be written in full when used for the first time.
Response: The abbreviations are defined in full at their first occurrence of the manuscript.

- The discussion is could be improved in my opinion and reference could be made to other publications. The authors do not go into depth about possible explanations of hypotheses for their findings. Why does a peptide that is believed to be cut out of the mature F protein elicit such a strong neutralizing antibody response? Similarly, if the p27 is still present in the F protein on the cell surface or in the F incorporated in virions, what would the consequences be for virus infectivity and the presence of other known neutralization sites? The p27 protein is heavily glycosylated with 3 N-glycans in this small peptide. A recent publication looked at the role of N-glycans in the p27 of RSV F and showed that removal of these glycosylations increase induction of neutralizing antibodies (Leemans et al., "Characterization of the role of N-glycosylation sites in the respiratory syncytial virus fusion protein in virus replication, syncytium formation and antigenicity". Virus Res. 2019 Jun;266:58-68."). Since N-glycans could be involved in 'glycan shielding) of the p27 peptide, this seems relevant to be discussed.

Response: As suggested by the reviewer, we have discussed our findings and expanded the results and discussion section for interpretation of the key observations.

Lines 154-173:

F-p27 is expressed on the surface of RSV-infected cells and in the lungs of RSV-infected mice

While p27 (residues 110-136) is not part of the mature F protein on virions, some immature or unprocessed F0 may be present on virions (Krzyzaniak, Zumstein et al., 2013) and might not be proteolytically activated until it reaches the target cells. On the other hand, p27 is part of newly translated F0 in RSV-infected cells. As can be seen in Fig. 4A, all mice vaccinated with the p27-containing peptide (110-136 residues) generated p27-peptide (110-136) antibody binding titers in ELISA but did not bind to F (1-34) peptide (negative control). Antisera from animals given "pre-F" or "post-F" proteins lacking the p27 sequence did not react to the p27 (110-136) peptide in ELISA.

To determine if the p27 peptide is expressed on the surface of RSV-infected cells, we generated rabbit antiserum against F protein and against F peptide containing residues 110-136 of p27 sequence. Immunostaining performed on non-permeabilized RSV A2-infected A549 cells demonstrated a comparable strong cell-surface staining patterns using either anti-RSV F protein or anti-F-p27 peptide antisera. Both antisera-stained similar percentages of RSV-A2 infected A549 cells (64.5% and 63.89 %, respectively) (Fig. 4B).

Furthermore, we also observed strong p27 expression in sections of lung tissues from RSV A2-infected animals using rabbit serum generated against either RSV F protein (62.25%) or F-p27 (110-136) peptide (61.59%) (Fig. 5). These observations suggest that F-p27-containing immature F0 is widely expressed on surface of RSV-infected cells in vitro and in RSV-infected lungs in vivo.

Lines 218-236:

The low binding of anti-p27 peptides to virions is explained by the fact that p27 is uniquely found in uncleaved F0 which is normally excised during F protein maturation into F1/F2 complex and is expected to be absent on mature RSV virion particles. This was partially explained by an early study demonstrating that the presence of p27 peptide has a destabilizing effect on trimer formation and incorporation into virions (Krarup et al., 2015). However, some immature (unprocessed F0) molecules may be found at low frequency on virions (Krzyzaniak et al., 2013), explaining the weak binding of anti-p27 serum to virions in ELISA.

In native F protein the p27 contains 2 out of five glycosylation sites found in the F protein (N116 and N126). However, Leemans et al., reported that infection of mice with recombinant virus lacking the N116 glycosylation site resulted in significantly higher neutralizing antibodies compared to wild-type RSV infection expressing fully glycosylated RSV-F (Leemans, Boeren et al., 2019). This finding further supports the hypothesis that fully glycosylated p27

is destabilizing the F trimer or interfere with proper folding of the F. In our study, the p27 peptide was unglycosylated (as chemically synthesized) and therefore could be more immunogenic than a fully glycosylated p27. Moreover, the antibodies elicited in mice or rabbits against p27 peptide stained both RSV infected cells in vitro and lung tissues from RSV challenged mice, suggesting that unglycosylated p27 peptide induced antibodies can recognize fully glycosylated p27 on RSV infected cells.

Lines 241-251:
However, minimal neutralization of RSV was measured in the RSV-LINT assay with sera from animals vaccinated with the p27 peptide. Therefore, the previously reported immunogenicity results with pre-fusogenic F RSV vaccine candidate containing p27 (Patel et al., 2019) showing higher neutralizing titers are unlikely due to anti-p27 antibodies contributing to the virus neutralization in vitro. The higher neutralizing activity of pre-fusogenic F RSV vaccine candidate is potentially due to other neutralizing sites within F as was demonstrated by generation of higher titer antibodies by pre-fusogenic F that competed with MAbs to site Ø, II, IV, and VIII compared with pre-fusion or post-fusion form of F (Patel et al., 2019). The study by Patel et al., also suggests that inclusion of p27 in a vaccine may not negatively impact development of antibodies away from neutralization sites (sites Ø -IV) of RSV-F.

Lines 267-284:
This study suggests that *in vivo* control of viral replication in RSV-infected animals may be manifested by multiple mechanisms such as Antibody-dependent cellular cytotoxicity (ADCC), cell-mediated immunity (CMI) and Fc-mediated binding and killing of RSV infected cells and immature virions expressing unprocessed F0, containing the p27 sequence, in addition to neutralization of cell free virions that express primarily mature F trimers. Staining of greater than 60% RSV-infected A549 cells and lung tissues post-RSV infection confirmed strong p27 cell surface expression (Figs. 4, 5). This suggests that a substantial number of RSV-infected cells express F0 in the immature form containing p27 *in vivo* on their surface, in agreement with the study by Krzyzaniak et al (Krzyzaniak et al., 2013). Such cells could be targeted by anti-p27 antibodies and subjected to killing via ADCC. We demonstrated that anti p27 antibodies mediate ADCC activity. Infected cells can also be targeted by cytotoxic T cells specific for p27-derived short peptides loaded on MHC molecules. We observed increase in the number of both CD4 and CD8 cells in lung tissues from RSV-infected (but not uninfected) mice. The numbers of T cells trended higher for vaccinated vs. PBS or KLH controls, however, they did not reach statistical significance, and were not particularly elevated in the p27 vaccinated animals compared with the other F-peptide vaccinated animals.

Methods:
- Line 225: I'm not sure if this freeze/thaw method is the most efficient method to prepare virus stocks, as this could result in a lot of debris in the virus preparation.

Response: The virus preparation undergoes several steps to remove any cell debris, including clarifying centrifugation and purification by sucrose-density gradient to obtain high quality pure virus preparations. See detailed methods:

Lines 305-310:

To generate a challenge virus stock, at 5 days post infection (dpi), cells were freeze-thawed twice, and virus was collected. Harvested viruses were cleared of cell debris by centrifugation at 3,795g for 15 min. Virus stocks used in challenge studies were pelleted by centrifugation at 10,509g overnight. Pelleted virus was resuspended in TNE buffer and purified by sucrose-gradient ultracentrifugation (Fuentes et al., 2017). Virus titers were determined by plaque assay on A549 cells. The optimal challenge dose (10^6 PFU/10 μL intranasally) was determined in an earlier study in which viral loads were measured by traditional plaque assay, by RT-qPCR, and by live imaging (flux) and gave comparable results in terms of viral kinetics and peak values (Fuentes et al., 2017).

- Line 249: The authors do not mention immunization of mice with a negative control in the methods-section, however this is described in the results-section. This should be added in the methods-section to be complete.

Response: We have added additional information in methods.

Lines 328-333:
Four- to 6-week-old female BALB/c mice (BALB/cAnNCr strain code #555) were obtained from Charles River Labs. Mice [N = 5 per group] were immunized intramuscularly (i.m.) at day 0 and day 28 with 20 μg of purified F protein or with 20 μg of KLH-conjugated peptides combined with Emulsigen adjuvant, or with PBS (no vaccination control), or with 100 μg of unconjugated KLH alone (carrier control). As live RSV-A2 virus control, a group of mice were given one intranasal dose of 10e4 pfu/10 μL virus.

- Line 254: When lung histopathology analysis is performed, anesthesia with isoflurane followed by CO2 asphyxiation can be of influence on cell counts afterwards. This has already been described.

Response: The anesthesia used in the entire study was cocktail of ketamine and dexmedetomidine. No isoflurane was used in this mice study. Methods

section has been rectified accordingly.

Lines 333-336: Blood was collected from the tail vein on days 0 and 35. On day 42, mice were anesthetized with ketamine and dexmedetomidine cocktail given intraperitoneally according to mouse body weight and infected intranasally (i.n.) with 10^6 PFU of rRSV-A2-L19-FFL as previously described (Fuentes et al., 2017).

Lines 381-382: The lung histopathology was performed after euthanizing mice with anesthetic cocktail containing ketamine and dexmedetomidine.

- Line 325: No permeabilization was performed with triton or other detergents. This might have been better for cytoplasmatic staining?

Response: Our intent was look at the cell-surface expression of F-p27 and therefore we did not permeabilize the cells. This has been clarified further:

Lines 409-410: Since we wanted to evaluate the presence of p27 on the cell surface, no cell permeabilization was performed.

Figure Legends:
- Line 451: It is not clear what is meant by 'in vivo imaging'. This is also not described in the methods-section.
Response: No in-vivo imaging was performed in this study. The sentence has been removed.

- Line 455: "...lung viral titers at day post-RSV challenge" Day is not specified.
Response: In figure 2, all samples evaluated were samples after second vaccination (day 35) prior to RSV challenge. This error has been corrected.

Lines 585-588
Figure 2. RSV binding and neutralizing antibody response following mice immunization.

Serum samples were collected at seven days after the second immunization from individual mice (day 35 from the start of the study) and were tested for RSV-A2 virion binding in ELISA and neutralization by an RSV-LINT assay against RSV A2 strain in A549 cells.

- Line 485-486: Incorrect/unclear syntax
Response: The error has been fixed in revised manuscript.

Lines 617-619: (A) Serum samples collected from individual mouse (M 1-5)

immunized with F 110-136 peptide were tested for antibody binding against F-p27 (110-136) peptide or F 1-34 peptide (control) in ELISA.

14th Oct 2021

Dear Dr. Khurana,

Thank you for the submission of your revised manuscript to EMBO Molecular Medicine, and please accept my apologies for the delay in getting back to you. We have now received feedback from 1 out of 2 reviewers whom we asked to re-evaluate your manuscript. As the referee #3 will unfortunately not be able to return his/her report in a timely manner we prefer to make a decision now in order to avoid further delay in the process. I am pleased to inform you that we will be able to accept your manuscript pending the following final amendments:

1) In the main manuscript file, please do the following:
- Correct/answer the track changes suggested by our data editors by working from the attached document.
- Limit keywords to max.5.
- Make sure that all special characters display well.
- Remove data not shown (p.9).
- In M&M, provide the antibody dilutions that were used for each antibody.
- In M&M, statistical paragraph should reflect all information that you have filled in the Authors Checklist, especially regarding randomization, blinding, replication etc.
- Correct the reference citation in the reference list. Where there are more than 10 authors on a paper, 10 will be listed, followed by "et al.". Please check "Author Guidelines" for more information.
https://www.embopress.org/page/journal/17574684/authorguide#referencesformat
2) Appendix: Please submit "Expanded View Content" file as an "Appendix" with table of content and rename the figure and the table to "Appendix Figure S1" and "Appendix Table S1", also in the main text.
3) Source data: Please submit one file per figure and name it "Source Data Figure 1" etc. (use a zip archive if multiple images need to be supplied for one Figure). Please check "Author Guidelines" for more information.
https://www.embopress.org/page/journal/17574684/authorguide#sourcedata
4) Synopsis:
- Synopsis image: Please provide a striking image or visual abstract as a high-resolution jpeg file 550 px-wide x (250-400)-px high to illustrate your article.
- Please check your synopsis text and image, revise them if necessary and submit their final versions with your revised manuscript. Please be aware that in the proof stage minor corrections only are allowed (e.g., typos).
5) For more information: There is space at the end of each article to list relevant web links for further consultation by our readers. Could you identify some relevant ones and provide such information as well? Some examples are patient associations, relevant databases, OMIM/proteins/genes links, author's websites, etc...
6) As part of the EMBO Publications transparent editorial process initiative (see our Editorial at http://embomolmed.embopress.org/content/2/9/329), EMBO Molecular Medicine will publish online a Review Process File (RPF) to accompany accepted manuscripts. This file will be published in conjunction with your paper and will include the anonymous referee reports, your point-by-point response and all pertinent correspondence relating to the manuscript. Let us know whether you agree with the publication of the RPF and as here, if you want to remove or not any figures from it prior to publication. Please note that the Authors checklist will be published at the end of the RPF.
7) Please provide a point-by-point letter INCLUDING my comments as well as the reviewer's reports and your detailed responses (as Word file).

I look forward to reading a new revised version of your manuscript as soon as possible.

Yours sincerely,

Zeljko Durdevic

***** Reviewer's comments *****

Referee #2 (Comments on Novelty/Model System for Author):

Mechanistic studies such as this one that improve our understanding of RSV inoculation and immunity are critical in providing insight that might lead to an efficacious RSV vaccine.

Referee #2 (Remarks for Author):

This is a full paper of science and not a short report. I have no further comments as the authors have done an admirable job responding to reviewers' criticisms

The authors performed the requested editorial changes.

Food and Drug Administration
Center for Biologics Evaluation and Research
Office of Vaccines Research and Review
Division of Viral Products

Dear Editor,

Thanks for accepting our manuscript for publication in *EMBO Molecular Medicine.* We would like to submit our revised manuscript EMM-2020-13847-V2 entitled '**PROTECTIVE ANTIGENIC SITES IDENTIFIED IN RESPIRATORY SYNCYTIAL VIRUS FUSION PROTEIN REVEALS IMPORTANCE OF p27 DOMAIN'** for publication in *EMBO Molecular Medicine*.

We thank Reviewer's for appreciating our study for publication without any further comments. We appreciate your editorial suggestions and from data editors and have addressed all of them in the revised manuscript as outlined below.

1) In the main manuscript file, please do the following:

- Correct/answer the track changes suggested by our data editors by working from the attached document.

Response: Added all information as required.

- Limit keywords to max.5.

Response: Reduced to 5.

- Make sure that all special characters display well.

Response: Checked.

- Remove data not shown (p.9).

Response: Removed.

- In M&M, provide the antibody dilutions that were used for each antibody.

Response: Added Ab dilutions in M&M.

- In M&M, statistical paragraph should reflect all information that you have filled in the Authors Checklist, especially regarding randomization, blinding, replication etc.

Response: Added all relevant information.

- Correct the reference citation in the reference list. Where there are more than 10 authors on a paper, 10 will be listed, followed by "et al.". Please check "Author Guidelines" for more information. https://www.embopress.org/page/journal/17574684/authorguide#referencesformat

Response: References formatted as suggested.

2) Appendix: Please submit "Expanded View Content" file as an "Appendix" with table of content and rename the figure and the table to "Appendix Figure S1" and "Appendix Table S1", also in the main text.

Response: Made relevant edits.

3) Source data: Please submit one file per figure and name it "Source Data Figure 1" etc. (use a zip archive if multiple images need to be supplied for one Figure). Please check "Author Guidelines" for more information. https://www.embopress.org/page/journal/17574684/authorguide#sourcedata

Response: Source data file are provided for each figure separately.

4) Synopsis:

- Synopsis image: Please provide a striking image or visual abstract as a high-resolution jpeg file 550 px-wide x (250-400)-px high to illustrate your article.

Response: Done

- Please check your synopsis text and image, revise them if necessary and submit their final versions with your revised manuscript. Please be aware that in the proof stage minor corrections only are allowed (e.g., typos).

Response: Done

5) For more information: There is space at the end of each article to list relevant web links for further consultation by our readers. Could you identify some relevant ones and provide such information as well? Some examples are patient associations, relevant databases, OMIM/proteins/genes links, author's websites, etc...

Response: No website links. Corresponding author can be reached by email.

6) As part of the EMBO Publications transparent editorial process initiative (see our Editorial at http://embomolmed.embopress.org/content/2/9/329), EMBO Molecular Medicine will publish online a Review Process File (RPF) to accompany accepted manuscripts. This file will be published in conjunction with your paper and will include the anonymous referee reports, your point-by-point response and all pertinent correspondence relating to the manuscript. Let us know whether you agree with the publication of the RPF and as here, if you want to remove or not any figures from it prior to publication. Please note that the Authors checklist will be published at the end of the RPF.

Response: We agree.

7) Please provide a point-by-point letter INCLUDING my comments as well as the reviewer's reports and your detailed responses (as Word file).

Response: Provided here.

We hope you will find the revised manuscript ready for publication in *EMBO Molecular Medicine.*

Sincerely,

Surender Khurana, Ph.D.

Center for Biologics Evaluation and Research (CBER)

US Food and Drug Administration (FDA)
Email: Surender.Khurana@fda.hhs.gov
Phone: 240-402-9632

We are pleased to inform you that your manuscript is accepted for publication and is now being sent to our publisher to be included in the next available issue of EMBO Molecular Medicine.

EMBO PRESS

YOU MUST COMPLETE ALL CELLS WITH A PINK BACKGROUND ➔

PLEASE NOTE THAT THIS CHECKLIST WILL BE PUBLISHED ALONGSIDE YOUR PAPER

Corresponding Author Name: SURENDER KHURANA
Journal Submitted to: EMBO
Manuscript Number: EMM-2020-13847-T

Reporting Checklist For Life Sciences Articles (Rev. June 2017)

This checklist is used to ensure good reporting standards and to improve the reproducibility of published results. These guidelines are consistent with the Principles and Guidelines for Reporting Preclinical Research issued by the NIH in 2014. Please follow the journal's authorship guidelines in preparing your manuscript.

A- Figures
1. Data
The data shown in figures should satisfy the following conditions:

➔ the data were obtained and processed according to the field's best practice and are presented to reflect the results of the experiments in an accurate and unbiased manner.
➔ figure panels include only data points, measurements or observations that can be compared to each other in a scientifically meaningful way.
➔ graphs include clearly labeled error bars for independent experiments and sample sizes. Unless justified, error bars should not be shown for technical replicates.
➔ if n< 5, the individual data points from each experiment should be plotted and any statistical test employed should be justified
➔ Source Data should be included to report the data underlying graphs. Please follow the guidelines set out in the author ship guidelines on Data Presentation.

2. Captions
Each figure caption should contain the following information, for each panel where they are relevant:

➔ a specification of the experimental system investigated (eg cell line, species name).
➔ the assay(s) and method(s) used to carry out the reported observations and measurements
➔ an explicit mention of the biological and chemical entity(ies) that are being measured.
➔ an explicit mention of the biological and chemical entity(ies) that are altered/varied/perturbed in a controlled manner.

➔ the exact sample size (n) for each experimental group/condition, given as a number, not a range;
➔ a description of the sample collection allowing the reader to understand whether the samples represent technical or biological replicates (including how many animals, litters, cultures, etc.).
➔ a statement of how many times the experiment shown was independently replicated in the laboratory.
➔ definitions of statistical methods and measures:
- common tests, such as t-test (please specify whether paired vs. unpaired), simple χ2 tests, Wilcoxon and Mann-Whitney tests, can be unambiguously identified by name only, but more complex techniques should be described in the methods section;
- are tests one-sided or two-sided?
- are there adjustments for multiple comparisons?
- exact statistical test results, e.g., P values = x but not P values < x;
- definition of 'center values' as median or average;
- definition of error bars as s.d. or s.e.m.

Any descriptions too long for the figure legend should be included in the methods section and/or with the source data.

In the pink boxes below, please ensure that the answers to the following questions are reported in the manuscript itself. Every question should be answered. If the question is not relevant to your research, please write NA (non applicable). We encourage you to include a specific subsection in the methods section for statistics, reagents, animal models and human subjects.

USEFUL LINKS FOR COMPLETING THIS FORM

http://www.antibodypedia.com
http://1degreebio.org
http://www.equator-network.org/reporting-guidelines/improving-bioscience-research-repor
http://grants.nih.gov/grants/olaw/olaw.htm
http://www.mrc.ac.uk/Ourresearch/Ethicsresearchguidance/Useofanimals/index.htm
http://ClinicalTrials.gov
http://www.consort-statement.org
http://www.consort-statement.org/checklists/view/32-consort/66-title
http://www.equator-network.org/reporting-guidelines/reporting-recommendations-for-tumo
http://datadryad.org
http://figshare.com
http://www.ncbi.nlm.nih.gov/gap
http://www.ebi.ac.uk/ega
http://biomodels.net/
http://biomodels.net/miriam/
http://jjj.biochem.sun.ac.za
https://osp.od.nih.gov/biosafety-biosecurity-and-emerging-biotechnology/
http://www.selectagents.gov/

B- Statistics and general methods

Please fill out these boxes ➔ (Do not worry if you cannot see all your text once you press return)

1.a. How was the sample size chosen to ensure adequate power to detect a pre-specified effect size?	Page 22
1.b. For animal studies, include a statement about sample size estimate even if no statistical methods were used.	Page 22
2. Describe inclusion/exclusion criteria if samples or animals were excluded from the analysis. Were the criteria pre-established?	Page 22
3. Were any steps taken to minimize the effects of subjective bias when allocating animals/samples to treatment (e.g. randomization procedure)? If yes, please describe.	Page 22
For animal studies, include a statement about randomization even if no randomization was used.	Page 22
4.a. Were any steps taken to minimize the effects of subjective bias during group allocation or/and when assessing results (e.g. blinding of the investigator)? If yes please describe.	Page 22
4.b. For animal studies, include a statement about blinding even if no blinding was done	Page 22
5. For every figure, are statistical tests justified as appropriate?	Page 22
Do the data meet the assumptions of the tests (e.g., normal distribution)? Describe any methods used to assess it.	Page 22
Is there an estimate of variation within each group of data?	Page 22

Is the variance similar between the groups that are being statistically compared?	Page 22

C- Reagents

6. To show that antibodies were profiled for use in the system under study (assay and species), provide a citation, catalog number and/or clone number, supplementary information or reference to an antibody validation profile. e.g., Antibodypedia (see link list at top right), 1DegreeBio (see link list at top right).	Pages 18-21
7. Identify the source of cell lines and report if they were recently authenticated (e.g., by STR profiling) and tested for mycoplasma contamination.	Pages 17 and 19

* for all hyperlinks, please see the table at the top right of the document

D- Animal Models

8. Report species, strain, gender, age of animals and genetic modification status where applicable. Please detail housing and husbandry conditions and the source of animals.	Page 16
9. For experiments involving live vertebrates, include a statement of compliance with ethical regulations and identify the committee(s) approving the experiments.	Page 22
10. We recommend consulting the ARRIVE guidelines (see link list at top right) (PLoS Biol. 8(6), e1000412, 2010) to ensure that other relevant aspects of animal studies are adequately reported. See author guidelines, under 'Reporting Guidelines'. See also: NIH (see link list at top right) and MRC (see link list at top right) recommendations. Please confirm compliance.	Page 22

E- Human Subjects

11. Identify the committee(s) approving the study protocol.	Not applicable
12. Include a statement confirming that informed consent was obtained from all subjects and that the experiments conformed to the principles set out in the WMA Declaration of Helsinki and the Department of Health and Human Services Belmont Report.	Not applicable
13. For publication of patient photos, include a statement confirming that consent to publish was obtained.	Not applicable
14. Report any restrictions on the availability (and/or on the use) of human data or samples.	Not applicable
15. Report the clinical trial registration number (at ClinicalTrials.gov or equivalent), where applicable.	Not applicable
16. For phase II and III randomized controlled trials, please refer to the CONSORT flow diagram (see link list at top right) and submit the CONSORT checklist (see link list at top right) with your submission. See author guidelines, under 'Reporting Guidelines'. Please confirm you have submitted this list.	Not applicable
17. For tumor marker prognostic studies, we recommend that you follow the REMARK reporting guidelines (see link list at top right). See author guidelines, under 'Reporting Guidelines'. Please confirm you have followed these guidelines.	Not applicable

F- Data Accessibility

18: Provide a "Data Availability" section at the end of the Materials & Methods, listing the accession codes for data generated in this study and deposited in a public database (e.g. RNA-Seq data: Gene Expression Omnibus GSE39462, Proteomics data: PRIDE PXD000208 etc.) Please refer to our author guidelines for 'Data Deposition'.<br><br>Data deposition in a public repository is mandatory for:<br>a. Protein, DNA and RNA sequences<br>b. Macromolecular structures<br>c. Crystallographic data for small molecules<br>d. Functional genomics data<br>e. Proteomics and molecular interactions	Page 23
19. Deposition is strongly recommended for any datasets that are central and integral to the study; please consider the journal's data policy. If no structured public repository exists for a given data type, we encourage the provision of datasets in the manuscript as a Supplementary Document (see author guidelines under 'Expanded View' or in unstructured repositories such as Dryad (see link list at top right) or Figshare (see link list at top right).	Not applicable
20. Access to human clinical and genomic datasets should be provided with as few restrictions as possible while respecting ethical obligations to the patients and relevant medical and legal issues. If practically possible and compatible with the individual consent agreement used in the study, such data should be deposited in one of the major public access-controlled repositories such as dbGAP (see link list at top right) or EGA (see link list at top right).	Not applicable
21. Computational models that are central and integral to a study should be shared without restrictions and provided in a machine-readable form. The relevant accession numbers or links should be provided. When possible, standardized format (SBML, CellML) should be used instead of scripts (e.g. MATLAB). Authors are strongly encouraged to follow the MIRIAM guidelines (see link list at top right) and deposit their model in a public database such as Biomodels (see link list at top right) or JWS Online (see link list at top right). If computer source code is provided with the paper, it should be deposited in a public repository or included in supplementary information.	Not applicable

G- Dual use research of concern

22. Could your study fall under dual use research restrictions? Please check biosecurity documents (see link list at top right) and list of select agents and toxins (APHIS/CDC) (see link list at top right). According to our biosecurity guidelines, provide a statement only if it could.	No
